# Metabolic engineering to enhance biosynthesis of both docosahexaenoic acid and odd-chain fatty acids in *Schizochytrium* sp. S31

**DOI:** 10.1186/s13068-019-1484-x

**Published:** 2019-06-08

**Authors:** Fangzhong Wang, Yali Bi, Jinjin Diao, Mingming Lv, Jinyu Cui, Lei Chen, Weiwen Zhang

**Affiliations:** 1 Center for Biosafety Research and Strategy, Tianjin University, Tianjin, People’s Republic of China; 2 Laboratory of Synthetic Microbiology, School of Chemical Engineering & Technology, Tianjin University, Tianjin, 300072 People’s Republic of China; 3 Frontier Science Center for Synthetic Biology and Key Laboratory of Systems Bioengineering (MOE), School of Chemical Engineering and Technology, Tianjin University, Tianjin, 300350 People’s Republic of China; 4 SynBio Research Platform, Collaborative Innovation Center of Chemical Science and Engineering, Tianjin University, Tianjin, 300350 People’s Republic of China

**Keywords:** Docosahexaenoic acid, Odd-chain fatty acids, Malic enzyme, Acetyl-CoA carboxylase, *Schizochytrium* sp. S31

## Abstract

**Background:**

Docosahexaenoic acid (DHA, C22:6) and odd-chain fatty acids (OCFAs, C15:0 and C17:0) have attracted great interest, since they have been widely used in food and therapeutic industries, as well as chemical industry, such as biodiesel production and improvement. The oil-producing heterotrophic microalgae *Schizochytrium* sp. 31 is one of main DHA-producing strains. Recently, it was found that *Schizochytrium* can also synthesize OCFAs; however, contents and titers of DHA and OCFAs in *Schizochytrium* are still low, which limit its practical application.

**Results:**

In this study, we found that acetyl-CoA carboxylase suffered from a feedback inhibition by C16-CoA in *Schizochytrium*, and relief of the inhibition resulted in improved both lipid content and the ratio of OCFAs in total fatty acids. Based on this finding, a novel strategy for elevating both DHA and OCFAs contents was established. First, the total lipid accumulation was increased by overexpressing a malic enzyme from *Crypthecodinium cohnii* to elevate NADPH supply. Second, the inhibition effect on acetyl-CoA carboxylase was relieved by overexpressing a codon-optimized *ELO3* gene from *Mortierella alpina*, which encodes an elongase enzyme responsible for converting C16 into C18 fatty acids. After the above two-step engineering, contents of DHA and OCFAs were increased by 1.39- and 3.30-fold, reaching a level of 26.70 and 25.08% of dry cell weight, respectively, which are the highest contents reported so far for *Schizochytrium*. The titers of DHA and OCFAs were elevated by 1.08- and 2.57-fold, reaching a level of 3.54 and 3.32 g/L, respectively. Notably, the OCFAs titer achieved was 2.66-fold higher than the highest reported in *Escherichia coli* (1.25 g/L), implying potential value for industry application. To reveal the potential metabolic mechanism for the enhanced biosynthesis of both DHA and OCFAs, LC–MS metabolomic analysis was employed and the results showed that the pentose phosphate pathway and the glycolysis pathway were strengthened and intracellular propionyl-CoA concentration were also significantly increased in the engineered *Schizochytrium*, suggesting an increased supply of NADPH, acetyl-CoA, and propionyl-CoA for DHA and OCFAs accumulation.

**Conclusions:**

The discovery provides a new source of OCFAs production, and proposes a new strategy to improve contents and titers of both DHA and OCFAs in *Schizochytrium*. These will be valuable for improving commercial potential of *Schizochytrium* and guiding the engineering strategy in other fatty acids producing heterotrophic microalga.

**Electronic supplementary material:**

The online version of this article (10.1186/s13068-019-1484-x) contains supplementary material, which is available to authorized users.

## Background

Docosahexaenoic acid (DHA, C22:6) and odd-chain fatty acids (OCFAs, C15:0, and C17:0) have attracted significant attentions because of various practical applications. DHA is an essential fatty acid that cannot be synthesized by human. It has been reported that DHA plays an important role in the development of nervous and visual systems in infants and young children. And it also has the positive effects on decreasing the risk of cardiovascular disease. Like DHA, OCFAs are also beneficial to human health. It is reported that OCFAs have clear association with lower multiple sclerosis, coronary heart disease, and type II diabetes risk [1–5]. Meanwhile, OCFAs have various important industrial applications, such as the precursors of plasticizers, herbicides, pharmaceuticals, and fragrance intermediates [6–9]. Furthermore, adding OCFAs or their derivates into biodiesels can be helpful for further improving quality of biodiesels [9–12]. OCFAs are important in making ruminant products as they have lower melting points, and higher concentration of OCFAs can make fat softer in lambs [13].

The industrial production of DHA has been carried out by heterotrophic microalgae fermentation, such as *Schizochytrium* and *Crypthecodinium* [14, 15]. Due to the difficulty related to genetic manipulation in the microalga, most of previous work has focused on optimizing fermentation condition and strain selection. For example, it was reported that 28.93 g/L of DHA and 151.40 g/L of biomass were achieved by optimizing oxygen transfer in *Schizochytrium* sp. S31 [16].

In recent years, various strategies have been adopted to enhance DHA production in microalga. For example, the DHA productivity of *Crypthecodinium cohnii* was doubled using a two-step chemical modulators based on adaptive laboratory evolution strategy [17], and *Agrobacterium*-mediated transformation, electroporation, and particle bombardment have been established in *Schizochytrium* sp. TIO1101, *Schizochytrium* sp. PKU#Mn4, and *Schizochytrium* sp. 31, respectively [18–20]. Although the efficiency is still low, metabolic engineering of *Schizochytrium* sp. to improve DHA accumulation has been gradually carried out [20–22]. For example, overexpression of anti-oxidative gene superoxide dismutase in *Schizochytrium* sp. PKU#Mn4 increased polyunsaturated fatty acids production by 1.37-fold [20].

Despite relatively few studies on OCFAs production were conducted so far, several strategies have been evaluated and the engineering of traditional fatty acid biosynthesis pathway is widely considered as an efficient strategy. For example, 1205 mg/L OCFAs were attained in *Escherichia coli* by increasing intracellular propionyl-CoA concentration and replacing enzymes involved in the initialization step of fatty acid elongation by enzymes with higher propionyl-CoA affinity [9]. Blocking competitive and degradation pathway and enhancing the downstream pathway are also used for OCFAs or derivatives production. In *Yarrowia lipolytica*, the ratio of OCFAs to total lipid was increased 1.65-fold by blocking the catabolic pathway of propionyl-CoA. Further genetic engineering by blocking β-oxidation, degradation of triacylglycerols, and pushing triacylglycerols biosynthesis, OCFAs content in dry cell weight was increased by 5.34-fold [12]. OCFAs or their derivatives can be manufactured by directly oxidation of even chain fatty acids. Cao et al. reported that the odd-chain fatty aldehydes can be formed by incorporating α-dioxygenase from *Oryza sativa* into *E*. *coli* to directly oxidize even chain C_n_ fatty acids into odd-chain C_n-1_ fatty acids [23]. In addition to above-mentioned strategies, other novel methods have been developed for odd-chain molecules synthesis. For example, the *E*. *coli* can produce C7, C9, and C11 fatty alcohols by reversal of the beta-oxidation cycles with supplement of propionate in the medium [24]. By adaptation of the butanol biosynthetic pathway and development of a complementary modular toolkit, *E*. *coli* can be engineered to synthesize various odd-chain products in a controlled manner [25].

The oleaginous heterotrophic microalgae *Schizochytrium* sp. S31 has received much attention as it can achieve high biomass accumulation with high percent of oil and DHA content. Until now, the highest yield and productivity of DHA were achieved using *Schizochytrium* sp. S31 [16]. Actually, it has been successfully applied for industrial production of DHA in several countries [14, 16, 26]. Recently, it was also found that the proportion of OCFAs in total fatty acids of *Schizochytrium* sp. S31 was much higher than those of other *Schizochytrium* strains [26–29]. Therefore, we propose that *Schizochytrium* sp. S31 could be an ideal candidate for producing both DHA and OCFAs. Such effort will not only provide a novel source for the production of OCFAs, but also improve commercial and industrial potential of *Schizochytrium* sp. S31.

In this study, inhibition effect on acetyl-CoA carboxylase activity was observed in *Schizochytrium*, and converting C16 into C18 fatty acids could relieve the inhibition effect, resulting in elevated content of total lipid and the ratio of OCFAs in total fatty acids. Based on the finding, a novel strategy for elevation of both DHA and OCFAs content was successfully applied in *Schizochytrium*. First, the total lipid accumulation was increased by reinforcing NADPH supply through overexpression of a malic enzyme. Second, coding gene of an elongase enzyme responsible for converting C16 into C18 was overexpressed to relieve the inhibition effect on acetyl-CoA carboxylase activity. After the modification, contents and titers of both DHA and OCFAs were significantly enhanced. The study also provides useful information to engineer microalgae for other high-valued fatty acid production.

## Results

### Lipid and OCFAs contents increased by overexpression of *ELO3* in *Schizochytrium*

**Fig. 1:**
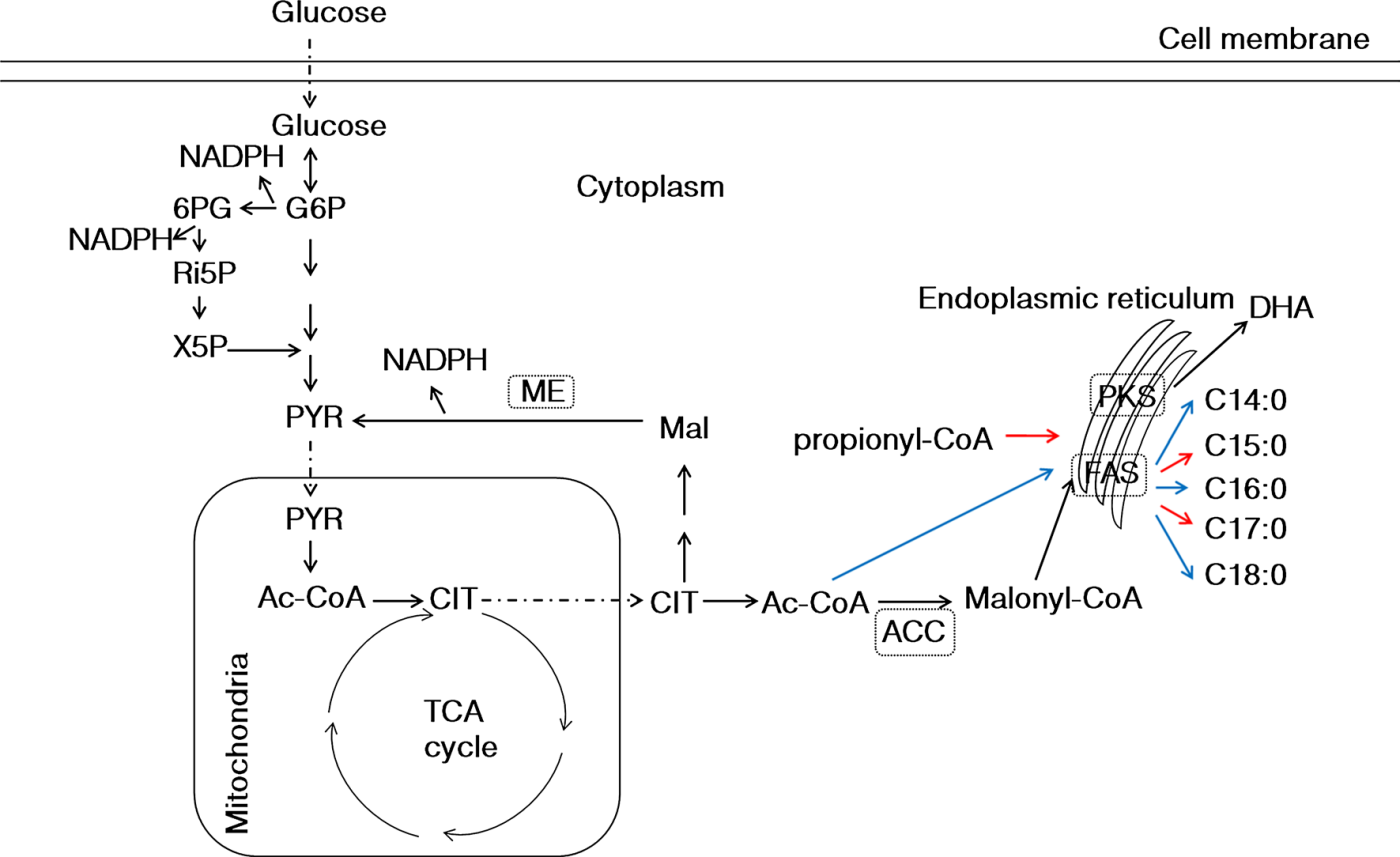
Schematic diagram of lipid biosynthesis pathway in *Schizochytrium* sp. S31. G6P, glucose-6-phosphate; 6PG, 6-phospho gluconate; Ri5P, ribose 5-phosphate; X5P, xylulose 5-phosphate; PYR: pyruvate; Ac-CoA, acetyl-CoA; CIT, citrate; MAL, malate; ME, malic enzyme; ACC, acetyl-CoA carboxylase; FAS, fatty acid synthase; PKS, polyketide synthase; DHA, docosahexaenoic acid; NADPH, nicotinamide adenine dinucleotide phosphate

Feedback inhibition of enzymes controlling the early steps of biosynthetic pathways was well observed in many microorganisms when the final products were accumulated at large quantitation [30, 31]. Early reports showed that C16-CoA (a kind form of C16:0) was able to feedback inhibit activity of acetyl-CoA carboxylase (ACC), an enzyme controlling the first committed step of fatty acid biosynthesis in many organisms (Fig. 1) [32]. Meanwhile, a previous study found that the content of C16:0 fatty acids in dry cell weight was as high as approximately 12.79% in *Schizochytrium* [29], which prompted us to explore whether the similar feedback inhibition mechanism is existing. The cell-free extract of *Schizochytrium* sp. 31 was prepared. The 50 or 100 μM of C16-CoA was added into the cell-free extraction and then acetyl-CoA carboxylase activity was determined. The results showed that acetyl-CoA carboxylase activity was decreased 38.24% or 44.98% in the presence of 50 or 100 μM of C16-CoA, respectively (Additional file 1: Fig. S1). Therefore, we concluded that acetyl-CoA carboxylase suffered from inhibition by C16-CoA.

A codon-optimized *ELO3* gene derived from *Mortierella alpina* has been well characterized with a high activity of converting C16 into C18 [33, 34]. Recently, it was also used for improving eicosapentaenoic acid (EPA, an omega-3 fatty acid) production in *Yarrowia lipolytica* [35]. ELO3 is carbon chain length-specific enzyme, as the previous study showed that it can efficiently convert C16:0 into C18:0, but cannot convert C17:0 into C19:0 [33]. Therefore, *ELO3* gene was chosen and transformed into *Schizochytrium* sp. S31 via electroporation. The schematic map of the *ELO3* expression cassette was shown in Additional file 1: Fig. S2A. After two rounds of neomycin selections, transformants were confirmed by PCR, as the PCR analysis of a positive transformant named S-E strain showed a 1.51 kb expected PCR band using primer 1 and 2, while no band was presented in the wild type (Additional file 1: Fig. S2B). Correspondingly, the transcript of the *ELO3* gene was successfully detected in the S-E strain, but not in the wild type (Additional file 1: Fig. S2C), demonstrating that *ELO3* gene has been successfully transformed into *Schizochytrium*.

**Fig. 2:**
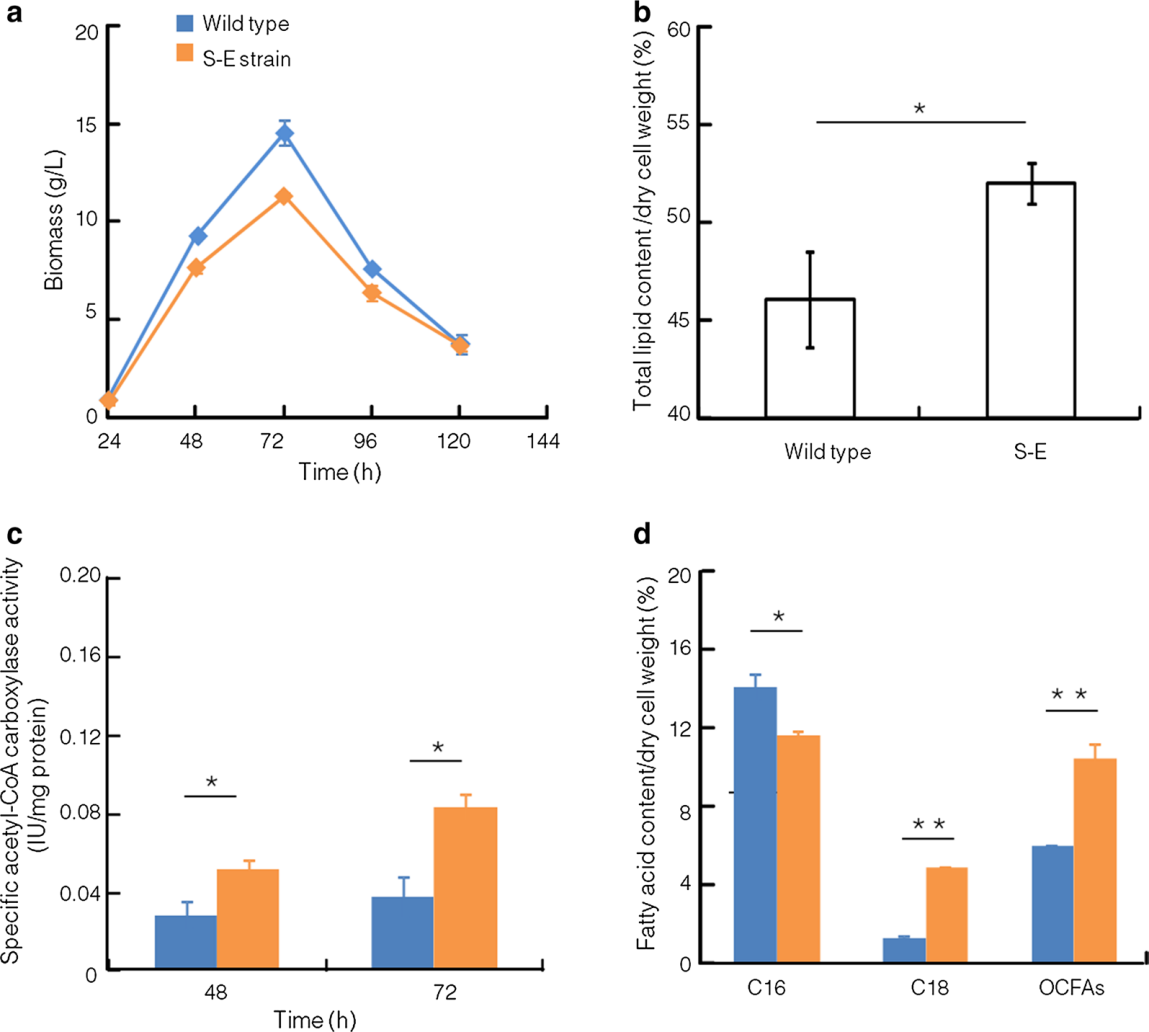
Comparison of growth curves, lipid contents and acetyl-CoA carboxylase activity between wild type and S-E. **a** Growth curves; **b** lipid content; **c** acetyl-CoA carboxylase activity; **d** fatty acid contents. blue: wild type; orange: S-E strain. *p < 0.05; **p < 0.01. C14, C14:0; C16, C16:0; C18, C18:0 and C18:1; OCFAs, odd-chain fatty acids (C15:0 + C17:0)

Analysis showed that the lipid content was increased by 12.87% at 72 h (Fig. 2b), although the growth rate and highest biomass accumulation of S-E strain were slightly lower than those of the wild type (Fig. 2a). And the activity of acetyl-CoA carboxylase was significantly up-regulated at 48 and 72 h in S-E strain (*p *< 0.05, *n* = 3) (Fig. 2c). Therefore, it seemed more carbon source was directed into lipid biosynthesis by up-regulating acetyl-CoA carboxylase activity (Fig. 1). Further analysis showed that similar to the change of lipid content, the contents of C18 fatty acids were also significantly increased, which was up-regulated by 3.82-fold. However, the C16:0 content was decreased by 17.30% at 72 h (Fig. 2d), which may be due to the increased conversion and carbon flow of C16 into C18 by *ELO3* in S-E strain. These results suggested that feedback inhibition on acetyl-CoA carboxylase could be partially released by expressing *ELO3* gene in *Schizochytrium* sp. S31. Interestingly, the ratio of OCFAs in total fatty acids was increased by 27.42% in S-E strain (Additional file 2: Table S1).

### Lipid content increased by overexpressing a malic enzyme gene from *C. cohnii* in *Schizochytrium*

It was previously reported that the total lipid content of *Schizochytrium* sp. HX-308 was increased by 15% after adding 4 g/L malic acid to elevate malic enzyme activity at rapid lipid accumulation stage [36]. In addition, the content of total fatty acids was decreased by 50% after malic enzyme activity was inhibited, demonstrating the important role of the malic enzyme during lipid biosynthesis in *C*. *cohnii* [37]. Therefore, efforts were made to increase total lipid content by overexpressing a malic enzyme gene from *C*. *cohnii* in *Schizochytrium*. The expression cassette containing malic enzyme and neomycin resistance genes was transformed into *Schizochytrium* via electroporation to obtain S-M strain (Additional file 1: Fig. S3A). A 1.84 kb DNA band was detected in the PCR with the S-M strain, but not with the wild type (Additional file 1: Fig. S3B). RT-PCR showed that a malic enzyme fragment was successfully amplified from the cDNA of S-M strain; in contrast, no product was detected in the wild type (Additional file 1: Fig. S3C). To further confirm that the malic gene was expressed at the protein level, cells of S-M and wild-type strains were collected at 48 and 72 h during fermentation in the rich medium. The cell-free extracts were prepared to determinate the malic enzyme activity and NADPH content. The results showed that the malic enzyme activity of the S-M strain was 1.83- and 2.45-fold higher than that of the wild type, and NADPH content was increased 1.67- and 2.52-fold in the S-M strain at 48 and 72 h, respectively (Additional file 1: Fig. S4). These results indicated that the malic enzyme from *C*. *cohnii* has been successfully integrated into *Schizochytrium* genome and functionally expressed.

**Fig. 3:**
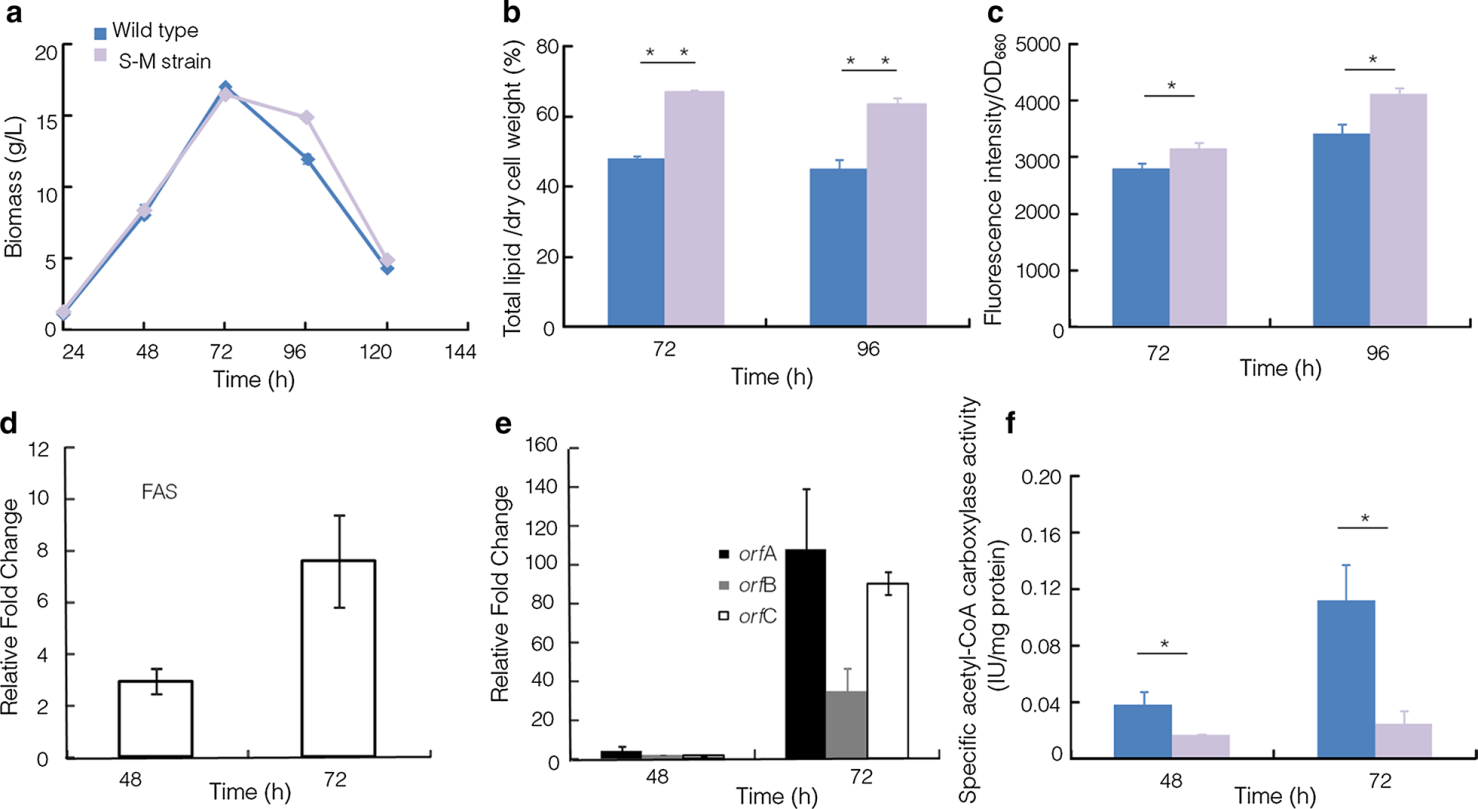
Comparison of growth, lipid content, and acetyl-CoA carboxylase activity between wild type and S-M strain. **a** Growth curves; **b** total lipid content; **c** the fluorescence intensity; **d** up-regulation of FAS in S-M strain; **e** up-regulation of *orf*A, *orf*B and *orf*C in S-M strain; **f** acetyl-CoA carboxylase activity. Blue: wild type; purple: S-M strain. *p < 0.05, **p < 0.01

As shown in Fig. 3a, the growth curves of two strains varied slightly, and the biomass accumulation reached the peak at 72 h of the fermentation and then quickly decreased. Cells were harvested at 72 and 96 h and then subjected to total lipid extraction analysis. As shown in Fig. 3b, the total lipid yield of the S-M strain was increased approximately 39.26% and 40.15%, reaching up to 67.16% and 63.71% in its cell dry weight at 72 and 96 h, respectively. Nile red staining analysis was also used to determine the neutral lipid accumulation. The fluorescence intensity of S-M strain was also significantly increased, comparing to the wild type (*p *< 0.05, *n* = 3) (Fig. 3c). Four genes associated with fatty acid biosynthesis were analyzed by qRT-PCR at 48 and 72 h. FAS gene, which is involved in saturate fatty acid biosynthesis, was up-regulated 2.92- and 7.55-fold in S-M strain (Fig. 3d), and three genes directly related to DHA biosynthesis, *orf*A, *orf*B, and *orf*C, were also significantly up-regulated (*p *< 0.05, *n* = 3) (Fig. 3e). These results suggested that overexpression of the malic gene from *C*. *cohnii* significantly promoted lipid synthesis; meanwhile, no compromise of cell growth was observed in the engineered *Schizochytrium*.

**Fig. 4:**
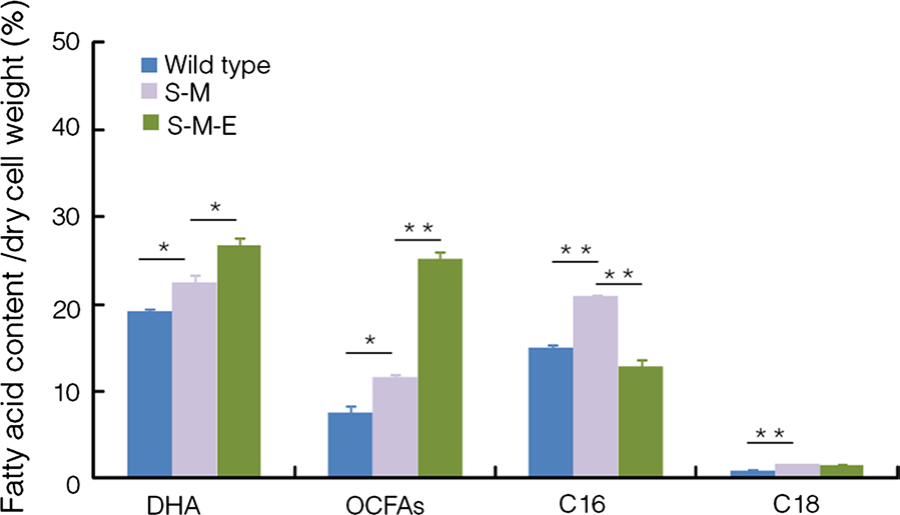
Comparison of the fatty acid contents among strains. Blue: wild type; purple: S-M strain; green: S-M-E strain. *p < 0.05. **p < 0.01. DHA, docosahexaenoic acid; C16, C16:0; C18, C18:0, and C18:1; OCFAs, odd-chain fatty acids (C15:0 + C17:0)

Compared to the wild type, the contents of DHA, OCFAs, C16:0, and C18 were increased 16.94%, 53.25%, 38.89%, and 75.37%, respectively in the S-M strain (Fig. 4), while the acetyl-CoA carboxylase activity was significantly decreased in the S-M strain at 48 and 72 h (*p *< 0.05, *n* = 3) (Fig. 3f). These results further confirmed that acetyl-CoA carboxylase suffered feedback inhibition in the S-M strain, and it is a potential metabolic engineering target for further enhancing DHA and OCFAs contents in *Schizochytrium* sp. S31.

### Elevated DHA and OCFAs contents by relieving inhibition effect on acetyl-CoA carboxylase in the S-M strain

**Fig. 5:**
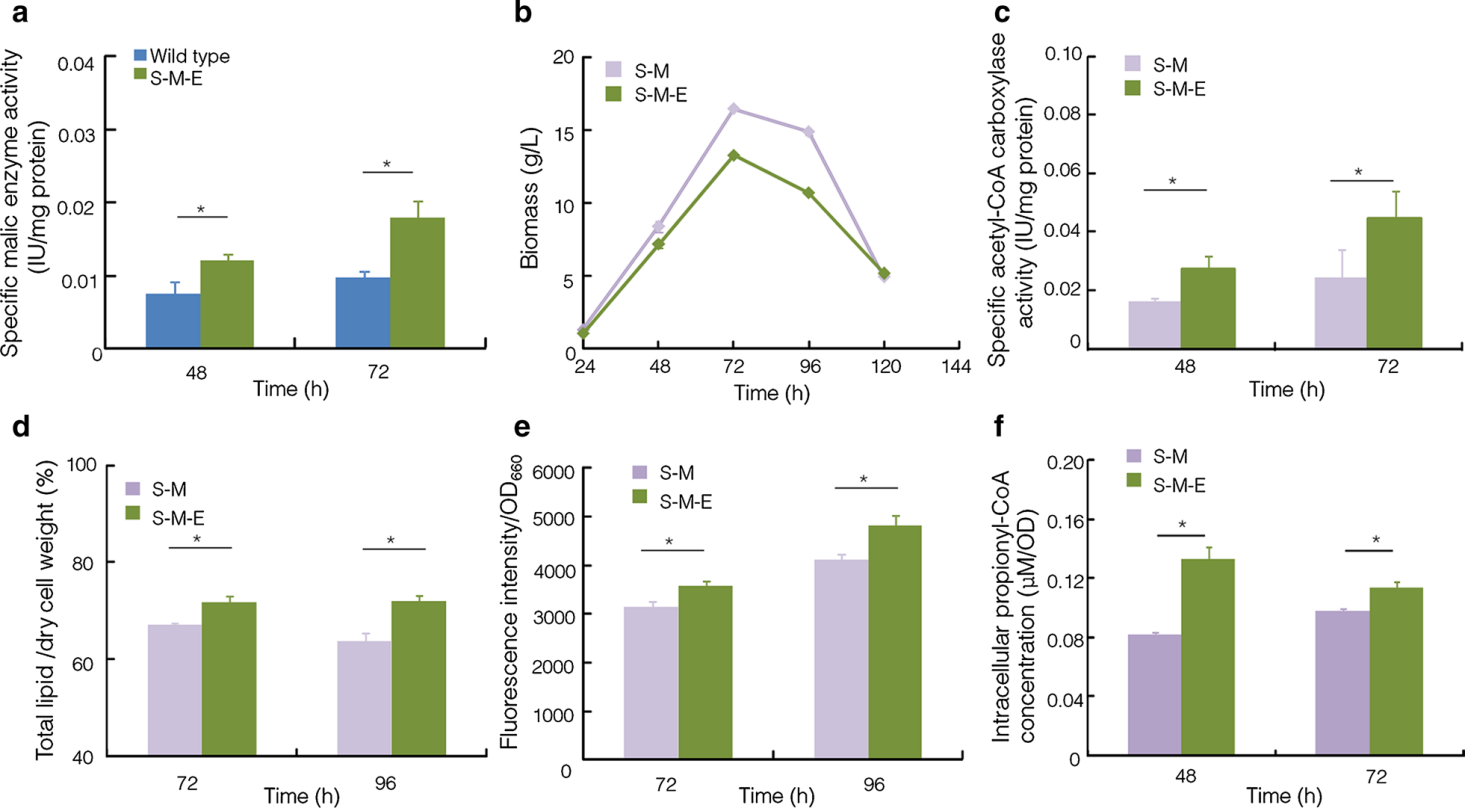
Comparison of enzyme activity, growth, lipid content and intracellular propionyl-CoA concentration among strains. **a** Malic enzyme activity; **b** growth curve; **c** acetyl-CoA carboxylase activity; **d** total lipid content; **e** the fluorescence intensity; **f** intracellular propionyl-CoA concentration. Blue: wild-type strain; purple: S-M strain; green: S-M-E strain. *p < 0.05, **p < 0.01

As mentioned above, overexpression of *ELO3* gene relieved inhibition effect on acetyl-CoA carboxylase in *Schizochytrium* sp. S31. Therefore, the expression cassette containing *ELO3* and *zeoR* was transformed into the S-M strain. A positive transformant named S-M-E strain was confirmed by both PCR and RT-PCR (Additional file 1: Fig. S5). Similar to the S-M strain, malic enzyme activity was significantly up-regulated in the S-M-E strain, in comparison with that in the wild type strain (Fig. 5a), suggesting that *ELO3* gene was successfully integrated into the S-M genome, and no interference on the function of the previously integrated malic gene was observed.

The S-M and S-M-E strains were in parallel inoculated into the rich fermentation medium. It was observed that the growth rate of S-M-E was slower than that of S-M. Both of them entered the stationary phase at the 72 h, but the highest biomass accumulation of the S-M-E was lower by 19.57% (Fig. 5b). The total lipid contents were also analyzed, and results showed a 6.73% or 12.97% increase of total lipid contents in the S-M-E strain at the 72 or 96 h, respectively, compared with the S-M strain (Fig. 5d). The fluorescence intensity of S-M-E strain was also significantly increased in comparison with that of S-M strain at the 72 and 96 h (*p *< 0.05, *n* = 3) (Fig. 5e). In addition, compared with the S-M strain, C16:0 content was decreased by 38.24%, and the contents of DHA and OCFAs were increased 1.19- and 2.16-fold in the S-M-E strain, respectively (Fig. 4). Furthermore, the acetyl-CoA carboxylase activity of S-M-E strain was significantly up-regulated at the 48 and 72 h (*p *< 0.05, *n* = 3) (Fig. 5c). Therefore, the feedback inhibition effect on acetyl-CoA carboxylase seemed relieved in the S-M-E strain.

**Fig. 6:**
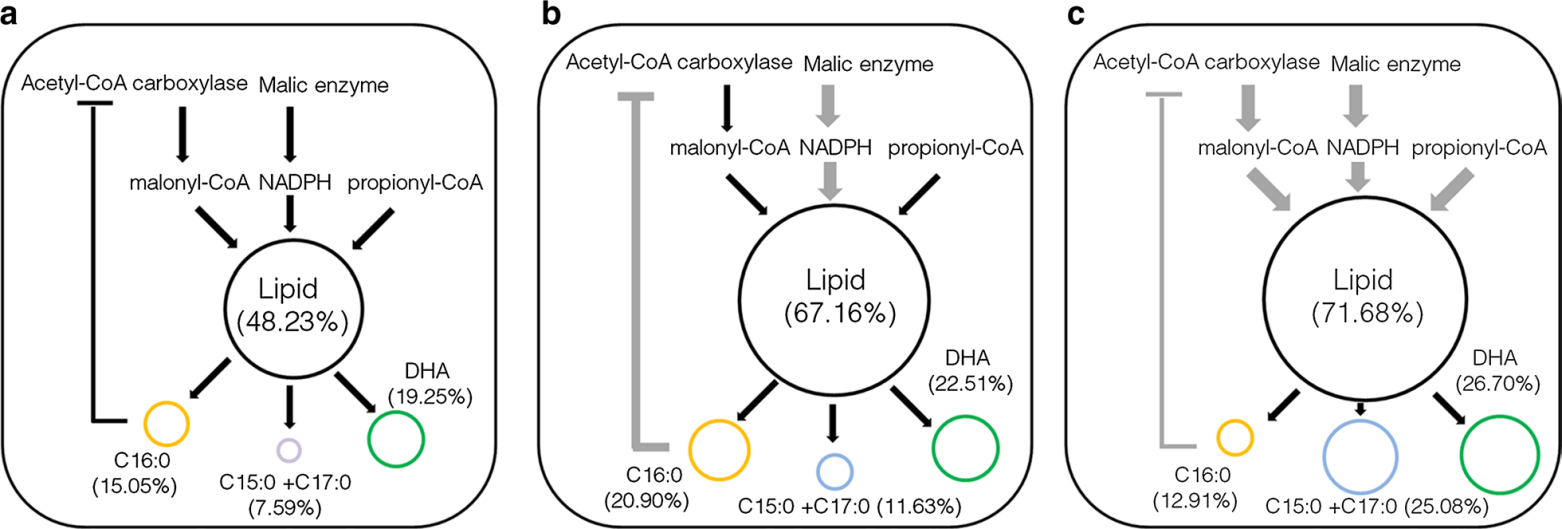
Schematic diagram of metabolic engineering strategy used in this study. **a** Wild-type strain; **b** S-M strain; **c** S-M-E strain. Black circle represents total lipid, yellow circle represents C16:0, blue circle represents OCFAs (C15:0 + C17:0), and green circle represents DHA. Black lines represent original pathway, and gray lines represent enhanced (bold) or decreased (slim) original pathways after engineering

**Fig. 7:**
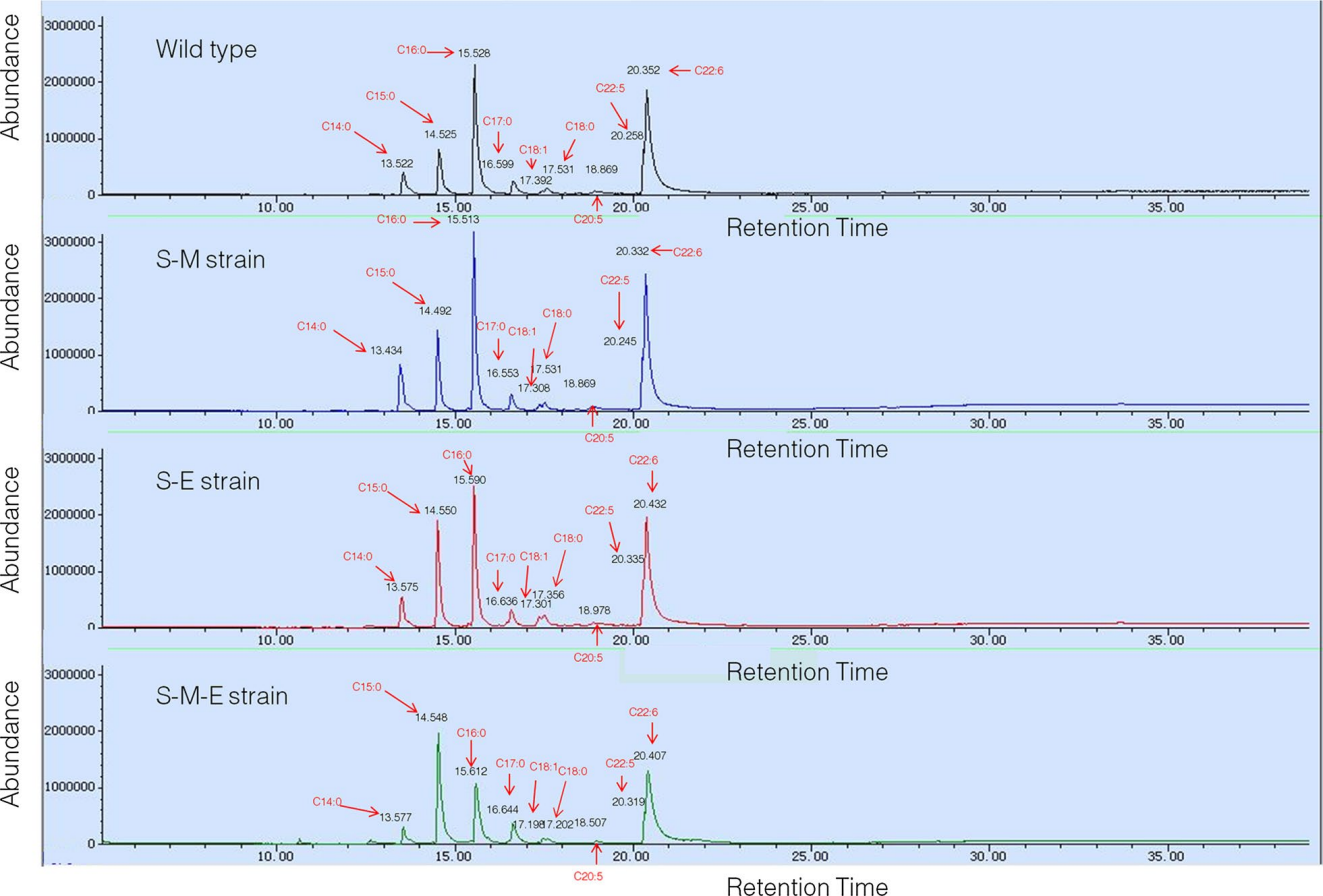
The GS/MS spectrums of methyl fatty acids profiles of all these strains. Black: wild type; blue: S-M strain; red: S-E strain; green: S-M-E strain

The schematic diagram of the changes of DHA, OCFAs, and C16:0 contents after two-step metabolic engineering is shown in Fig. 6. The GS/MS spectra of fatty acids profiles of all these strains are provided in Fig. 7 and Additional file 1: Fig. S6. The contents of lipids, DHA, and OCFAs were increased 1.49-, 1.39-, and 3.30-fold, reaching a level of 71.68, 26.70, and 25.08% of dry cell weight, in the final engineered *Schizochytrium* sp. S31. And the titers and yields of biomass, lipid, DHA, and OCFAs are summarized in Table 1. Notably, the OCFAs titer was 3.32 g/L, which was much higher than the highest report so far in *E*. *coli* (1.25 g/L) [9].

**Table 1:** Comparison of biomass, lipid, DHA, and OCFAs production in parent, S-M and S-M-E strains

	Biomass		Lipid		DHA		OCFAs	
	Titer (g/L)	Yield (g/g)	Titer (g/L)	Yield (g/g)	Titer (g/L)	Yield (g/g)	Titer (g/L)	Yield (g/g)
Parent	17.04 ± 0.19	0.43 ± 0.00	8.22 ± 0.13	0.21 ± 0.00	3.28 ± 0.09	0.08 ± 0.00	1.29 ± 0.11	0.03 ± 0.00
S-M	16.48 ± 0.11	0.41 ± 0.00	11.07 ± 0.02	0.28 ± 0.00	3.71 ± 0.14	0.09 ± 0.00	1.92 ± 0.05	0.05 ± 0.00
S-M-E	13.25 ± 0.02	0.33 ± 0.00	9.50 ± 0.19	0.24 ± 0.00	3.54 ± 0.11	0.09 ± 0.00	3.32 ± 0.14	0.08 ± 0.00

### Comparative metabolomics analysis of S-M and S-M-E strains

The LC–MS metabolomics have been established for quantifying metabolites related to central carbon metabolic pathway in microalgae previously in our laboratory [17, 38]. Therefore, it was applied to investigate possible mechanism related to enhanced accumulation of DHA and OCFAs in the S-M-E strain. Cells were harvested at 48 and 72 h, which are corresponding to exponential and stationary phases of the fermentation, and subjected to quantitative analysis of the targeted intracellular metabolites (Additional file 2: Table S2). The reliability of LC–MS metabolomics analysis was demonstrated by the principal component analysis. It was shown that three biological replicates of each sample were clearly clustered together, suggesting a good reproducibility (Additional file 1: Fig. S7). Meanwhile, the samples of S-M and S-M-E strains were visibly separated, suggesting that the metabolic change between the two strains can be detected by the analysis.

**Fig. 8:**
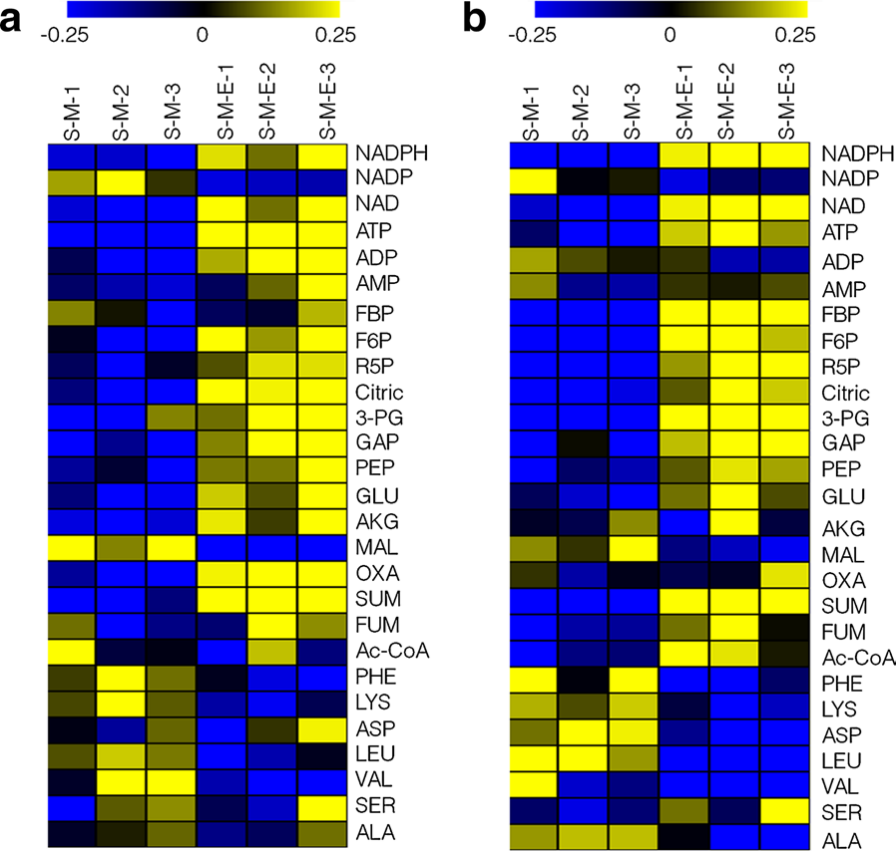
Heatmaps of LC–MS targeted metabolomics of S-M and S-M-E strains. **a** Heatmap at the 48 h; **b** Heatmap at the 72 h. NADPH, nicotinamide adenine dinucleotide phosphate; NADP+, oxidized form of nicotinamide adenine dinucleotide phosphate; NAD+, nicotinamide adenine dinucleotide; ATP, adenosine triphosphate; ADP, adenosine diphosphate; AMP, adenosine monophosphate; FBP, fructose 1,6-bisphosphate; F6P, fructose 6-phosphate; R5P, ribose 5-phosphate; Citric, citric acid; 3-PG, 3-phosphoglyceric acid; GAP, glyceraldehyde 3-phosphate; PEP, phosphoenolpyruvic acid; GLU: glutamic acid; AKG, 2-oxoglutaric acid; MAL: malate acid; OXA, oxaloacetic acid; SUM, succinic acid; FUM, fumaric acid; Ac-CoA, acetyl coenzyme A; PHE, phenylalanine; LYS, lysine; ASP, aspartic acid; LEU, isoleucine; VAL: valine; SER, serine; ALA, alanine

Heatmaps of targeted metabolite analysis in all samples were constructed by MultiExperiment Viewer software (Fig. 8). It was shown that, at 48 h, intercellular abundance of NADPH, NAD, ATP, ADP, F6P, R5P, Citric, GAP, PEP, GLU, AKG, OXA, and SUM was up-regulated, while intercellular abundance of NADP, MAl, PHE, LYS, LEU, and VAL was down-regulated in the S-M-E strain; at 72 h, intercellular abundance of NADPH, NAD, ATP, FBP, F6P, R5P, Citric, 3-PG, GAP, PEP, GLU, SUM, FUM, and Ac-CoA was up-regulated, and intercellular abundance of NADP, MAl, PHE, LYS, ASP, LEU, VAL, and ALA was down-regulated in S-M-E strain. Metabolic analysis of the metabolomic data showed that: (i) pentose phosphate pathway was up-regulated, as two key metabolites, GAP and R5P, were all up-regulated at both 48 and 72 h in the S-M-E strain. Pentose phosphate pathway is known to produce NADPH. In oleaginous yeast, up-regulation of pentose phosphate pathway resulted in significantly increase of lipid content, while, in *Aurantiochytrium* sp. SD116, strengthening pentose phosphate pathway increased the proportion of DHA in total lipids [39–41]; (ii) glycolysis pathway seemed strengthened as the up-regulation of FBP, F6P, 3-PG, and PEP was observed. The increased glycolysis pathway could lead to more Ac-CoA and ATP produced, which will further promote fatty acid accumulation [42, 43]; (iii) the lipid biosynthetic pathway seemed enhanced as the precursors of lipid biosynthesis, Ac-CoA and NADPH, was increased. We determined and compared the intracellular propionyl-CoA concentration between S-M and S-M-E strains, and the results showed that the propionyl-CoA concentration of S-M-E strain was significantly higher than that of S-M strains (Fig. 5f). In summary, we speculated that a possible mechanism responsible for increased DHA and OCFAs’ contents in S-M-E strain might be involved in up-regulation of pentose phosphate pathway, glycolysis pathway, and intracellular propionyl-CoA concentration to increase a supply of NADPH, acetyl-CoA, and propionyl-CoA for lipid biosynthesis.

## Discussion

Rational design of microbial factories has been widely used for production of high-value biochemicals. Recent studies on metabolic engineering for DHA production have mainly focused on *Schizochytrium* and *Crypthecodinium*, while *E*. *coli* and *Y*. *lipolytica* are usually used as hosts for OCFAs production. However, few reports focused on simultaneously producing high amounts of DHA and OCFAs in one microorganism. To maximize metabolic efficiency and potential value of *Schizochytrium* fermentation, we decided to produce both DHA and OCFAs simultaneously, as DHA and OCFAs can be separated and purified from the mixture by very established and low-cost method of urea complexation [44]. One of the main challenges in the industrial production of fatty acids from *Schizochytrium* is the high production cost [45, 46]. Compared with early reports [19–21], the strategy which we developed can significantly improve the contents and titers of DHA and OCFAs in *Schizochytrium*, especially the contents of DHA and OCFAs. Therefore, the total fatty acid production cost could be significantly decreased (Additional file 2: Table S3).

*Yarrowia lipolytica* and *E*. *coli* have been used for OCFAs production, and the exogenous propionate, which can be converted into propionyl-CoA, is supplemented into glucose medium to enhance intracellular propionyl-CoA concentration [9, 12]. Even so, the content of OCFAs in *Y*. *lipolytica* (2.30%, g/g dry cell weight, wild type) was still much lower than that in *Schizochytrium* (5.59%, g/g dry cell weight, wild type), which only used glucose as carbon source [12, 29]. For *E*. *coli,* in addition to above issues, a key defect is phage contamination, which could result in cell death, during industrial application, in comparison with other eukaryotic microorganisms [47]. *Schizochytrium* exhibited strong robustness and has already widely used for industrial production of DHA. The precursors of lipogenesis are abundant, since the oil content can reach roughly 50–55% in dry cell weight [16]. In this study, we did not add propionate into medium, but the OCFAs titer (3.32 g/L) in the final engineered strains was 2.66-fold higher than the highest report so far (1.25 g/L), which was achieved in the presence of propionate in *E*. *coli* [9]. Above all, *Schizochytrium* could be a new candidate for industrial production of OCFAs.

The primary source for lipogenic NADPH was varied in different species. In *Yarrowia lipolytica*, it was reported that pentose phosphate pathway is responsible for generating NADPH for lipogenesis, while malic enzyme might not be involved in lipid accumulation [39–41]. In our study, we found that both malic enzyme and pentose phosphate pathway are important to increase NADPH availability for lipid synthesis in *Schizochytrium* sp. S31. Although the genome of *Schizochytrium* sp. S31 has not been sequenced, the fatty acid pathways have been well characterized previously [48, 49]. The early studies showed that polyunsaturated fatty acids were synthesized by polyunsaturated fatty acid synthase (PUFA), while short or medium fatty acids (including OCFAs) were synthesized by fatty acid synthase (FAS). It has been proposed that NADPH produced by malic enzyme-involved transhydrogenase system was preferred for supporting biosynthesis of saturated fatty acid through the FAS pathway [37, 50–52], and NADPH produced by pentose phosphate pathway was closely involved in DHA production by PKS pathway [37, 50, 52, 53]. In this study, it was found that overexpression of malic enzyme was able to increase the ratio of C16:0 and OCFAs in total lipid and strengthened pentose phosphate pathway increased the percentage of DHA in total lipid (Fig. 6), consistent with the early conclusion [37, 50, 52, 53]. These results also suggested that regulation mechanisms of FAS and PKS pathways could be different, which may result in increase of DHA and OCFAs at different folds.

The lipogenesis begins by the action of acetyl-CoA carboxylase, and ends by an thioesterase for hydrolyzing C16- or C18-ACP into mature C16 or C18 fatty acids [51] (Fig. 1). To our knowledge, there is only one research on inhibition effect of acetyl-CoA carboxylase in microalga [32], but lacks evidence *in vivo* and methods for alleviating the inhibition effect. In our study, *in vitro* study showed that acetyl-CoA carboxylase suffered from inhibition by C16-CoA, and *in vivo* study showed that decreasing the content of C16 fatty acid was able to elevate acetyl-CoA carboxylase activity and increasing C16 fatty acid content decreased acetyl-CoA carboxylase activity. Therefore, we concluded that there exists a feedback inhibition effect on acetyl-CoA carboxylase by C16-CoA or other forms of C16 in *Schizochytrium* sp. S31. We also proposed a novel strategy to release the inhibition of acetyl-CoA carboxylase by overexpressing *ELO3* genes to convert C16 into C18, not by overexpressing thioesterase to hydrolyze fatty acid-ACP into free fatty acid.

Literature also pointed that the similar feedback inhibition mechanism might also exist in other microalgae. For example, although no increase of lipid content has been reported by overexpressing acetyl-CoA carboxylase in *C*. *reinhardtii* [54], overexpression of acyl-ACP thioesterase was found to efficiently improve lipid content [55], suggesting that overexpression of acetyl-CoA carboxylase gene could result in increased C16 or its intermediates contents and, correspondingly, a feedback inhibition of acetyl-CoA carboxylase activity, while overexpression of acyl-ACP thioesterase facilitated the subsequent conversion of C16 or its intermediates into triacylglycerols, leading to relief of the inhibition and the elevated lipid biosynthesis.

We also found that the up-regulation of OCFAs content was accompanied by the slight decrease of biomass accumulation. It is a common phenomenon observed previously in *E*. *coli* and *Y. lipolytica* [9, 12], but the underlying mechanism is unclear. LC–MS metabolomics suggested that Krebs cycle seemed not under-regulated in the S-M-E strain, suggesting not the metabolic burden that has caused the growth repression. It is an interesting question that we will further explore in the future.

## Conclusions

In this study, a two-step engineering strategy was established to increase the contents and titers of DHA and OCFAs in *Schizochytrium*. First, total lipid accumulation was increased by reinforcing NADPH supply. Second, the inhibition effect on acetyl-CoA carboxylase activity was relieved to further elevate lipid content and, meanwhile, improve the ratios of OCFAs in total fatty acids. After metabolic engineering, contents and titers of DHA and OCFAs achieved a level of 26.70 and 25.08% of dry cell weight, respectively, representing the highest contents in *Schizochytrium* so far. Furthermore, the final strain produced 3.32 g OCFAs/L medium, which was the highest titer reported so far. DHA and OCFAs are important health products and industrial intermediates. The efforts can improve commercial potential of *Schizochytrium*, and provide valuable reference for production of high-value products in microalga.

## Methods

### Strains and cultivation

*Schizochytrium* sp. S31 (ATCC 20888) was obtained from the American Type Culture Collection (MD, USA) and cultivated in a basal liquid medium contained 5.0 g/L glucose, 1.0 g/L yeast extract (OXOID, Basingstoke, UK), 1.0 g/L peptone, and 20.0 g/L sea salt (Sigma-Aldrich, St. Louis, MO, USA). The seed cultures were cultivated in basal liquid medium at 25 °C and shaken at 180 rpm for 48 h, and 5% (*v*/*v*) of seed cultures were transferred into rich fermentation medium containing 40 g/L glucose, 5 g/L peptone, 10 g/L yeast extract, and 20 g/L sea salt.

### Construction of gene expression cassette

All the plasmids were constructed using Seamless Cloning kit (Biomed, Beijing, China). CaMV35s promoter, CaMV poly(A) terminator and Nos terminator were amplified from the P1301-egfp plasmid. The 18s upstream, downstream, and α tubulin promoter were amplified from the *Schizochytrium* genome. The malic enzyme gene (GFIV01087707.1) was amplified from the cDNA of *C*. *cohnii* ATCC 30556. Neomycin resistance gene, bleomycin resistance gene, codon-optimized *Mortierella alpina ELO3* gene (Additional file 3: file S1), and CYC1 terminator were synthesized by Genewiz Company (Suzhou, China). The schematic maps of gene expression cassette are shown in Additional file 1: Figs. S1, S2 and S3. The gene expression cassette was ligated into the Pug6 plasmid and stored in − 20 °C until use.

### Transformation of *Schizochytrium*

Transformation of *Schizochytrium* was conducted following the method described previously with modification [56]. Ten milliliters of mid-log phase cells were harvested and washed using solution containing 5.0% PEG 8000 and 20 g/L sea salt for once. The cells were re-suspended in 0.4 mL of the same solution and added into a 1.5 mL microcentrifuge tube containing 250 mg of sterilized zirconia. The 1.5 mL microcentrifuge tubes were agitated at top speed on a Vortex Genie II mixer for 30 s to damage the cell walls. The treated cells were collected and washed with solution containing 20 g/L of sea salt for once and then with solution containing 50 mM sucrose for three times. Ten microliters of DNA (> 500 ng/µL) were used to transform 100 µL of treated cells by electroporation. The condition for electroporation was 2000 V field strength, 50 µF capacitance, 200 Ω resistance, and 2 mm cup. After electroporation, cells were recovered in 0.6 mL of the rich medium, and cultivated for 12 h at 25 °C and 180 rpm. Then, 200 µL of cell suspension were spread into basal medium plates containing antibiotics at suitable concentration. The concentrations of G418 and bleomycin were 100 µg/mL and 50 µg/mL, respectively. The plates were cultivated at 25 °C for 5 days, and transformants were transferred into the basal medium plates containing 600 µg/mL of G418 or 200 µg/mL of bleomycin. The transformants that were able to grow on plates containing high resistant concentration were picked and further confirmed by PCR and RT-PCR.

### Analyses of cell density, lipid content, fatty acid profile, and NADPH content

Cell density was determined by a UV-1750 spectrophotometer (Shimadzu, Japan) at OD_660_. For Nile Red analysis, 1.0 OD (OD_660_) cells were harvested, washed, and re-suspended in 1 mL of phosphate buffer saline (pH 7.2). After adding 10 μL Nile Red (10 mM), cells were incubated at 37 °C for 15 min and determined by a spectrophotometer (F-2700EL, HITACHI, Chiyoda, Japan). For total lipid content analysis, the method described previously was followed [29]. Briefly, total lipid of about 25.0 mg of lyophilized *Schizochytrium* algae powder was extracted by solutions containing chloroform: methanol (2:1, *v*/*v*) with 0.01% butylated hydroxytoluene for three times. The extracts were then washed with 1.0 mL of solution containing 1.0 M KCl for once, and with double-distilled water. The solvents were removed using a vacuum concentrator system (ZLS-1, Hunan, China). Fatty acid profile analysis was conducted following the method described previously [29]. Briefly, approximately 25 mg of lyophilized *Schizochytrium* algae powder was re-suspended in solutions containing 2 mL of methanol with 3% sulfuric acid and 2 mL of chloroform. The solutions were heated at 98 °C for 2 h. After cooling, 1 mL of distilled water was added and vortexed for 20 s. GC–MS experiments were performed using an Agilent 5975 MSD/7890 instrument (Agilent Corp, Santa Clara, CA, USA). The column was an HP-5MS (30 m × 0.25 mm × 0.25 μm film; Restek, Bellefonte, PA, USA). Ultra high purity helium was used as the carrier gas in a constant flow mode of 1 mL/min, and 1 μL of a given sample was injected in 1:2 split ratio mode. The temperature was kept at 80 °C for 2 min, and increased to 250 °C at a rate of 10 °C/min. When it reached 250 °C, the heating rate was decreased to 5 °C/min until 300 °C. The temperature was hold at 300 °C for 10 min before the analysis was terminated. NIST 11 mass spectral library (NIST/EPA/NIH mass spectral library, 2011 edition) was used for identified fatty acid methyl esters. The NADPH content was measured using Coenzyme II NADP(H) content determination kit (Nanjing Jiancheng Bioengineering Institute, Nanjing, China). Briefly, 7.5 OD of cells were harvested at 48 or 72 h, added with 1 mL of alkaline extract solution, and disrupted by cell disrupt apparatus. The solution was centrifuged at 14,000×*g* for 1 h. Five hundred microliters of supernatant was extracted and added with 500 μL of acid extract solution. The solution was centrifuged at 14,000×*g* for 10 min, and the supernatant extracted for NADPH content determination. The reaction agents were prepared according to the manufacture’s protocol. Distilled water was used as control. The absorbance wavelength was set at 570 nm. The equation for calculating NADPH content was: NADPH content (nmol/OD) = 0.0288 × (Δ*A*_(sample−control)_ − 0.072).

### RT-PCR and real-time PCR analyses

Cell were harvested and re-suspended in Trizol reagent (Invitrogen, Camarillo, CA, USA). The extraction of total RNA was conducted using a miRNeasy Mini Kit (Qiagen, Valencia, CA, USA), and subsequent synthesis of cDNA was achieved using a SuperScript^®^ III First-Strand Synthesis SuperMix kit (Thermo-Fisher, Waltham, MA, USA). The cDNA was used as the template for RT-PCR analysis with the primers listed in Additional file 2: Table S4. Real-time PCR reactions were performed using a StepOne Plus Real-Time PCR (Applied Biosystems, Foster City, CA, USA). The condition for real-time PCR was according to the protocol of UltraSYBR Mixture (CoWin Biotech. Co. Ltd., Beijing, China). 18S rRNA was used as an internal control. The relative transcription levels of FAS, *orf*A, *orf*B, and *orf*C genes were calculated using the 2^−ΔΔCt^ method [57]. The primers used for real-time PCR analysis are listed in Additional file 2: Table S4.

### Determination of enzyme activity

The 5.0 OD (OD_660_) of cells was collected at 48 and 72 h, and then washed using PBS buffer. The cell sediments were re-suspended in an extraction buffer (containing 100 mM KH_2_PO_4_/KOH (pH 7.5), 20% (v/v) glycerol), and disrupted by HNX-2 cell disruptor (Honour, Tianjin, China). The cell homogenates were centrifuged at 14,000×*g* (Eppendorf 5430R, Hamburg, Germany) for 30 min at 4 °C, and the supernatants were used for enzyme and protein content analysis. The protein concentration of the supernatants was determined by the Bradford protein assay using Coomassie Brilliant Blue G-250 (Solarbio, Beijing, China) according to the manufacturer’s instructions. The activity of NADP^+^-dependent malic enzyme was measured following the methods described previously [17]. Briefly, cell extract was added into the reaction solution containing 0.5 mM l-malate, 4 mM MnCl_2_, and 0.23 mM TPN in 66.67 mM triethanolamine buffer at pH 7.4, and incubated at 25 °C for 10 min. The NADPH formed was determined at 340 nm. The enzyme activity was defined as the conversion of 1.0 μM of l-malate and NADP to pyruvate, CO_2_ and NADPH per minute at pH 7.4 at 25 °C [17]. The activity of acetyl-CoA carboxylase was determined as described previously [58, 59]. Briefly, cell extract solution was added into a solution containing 1 mM acetyl-CoA, 15 mM KHCO_3_, 5 mM MnCl_2_, and 5 mM ATP, 1 mg/mL bovine serum albumin, and 3 mg/L biotin in 100 mM phosphate buffer at pH 8.0. The mixture was incubated at 30 °C, and equal aliquots were extracted every 2 min and immediately added 10% (w/v) trifluoroacetic acid to stop the reaction. The acetyl-CoA in each aliquot reacted with oxaloacetate to form citrate and CoA-SH. The CoA-SH was determined by reacting with dithiobisnitrobenzoic acid to form a yellow compound, which was assessed spectrophotometrically at 412 nm. The enzyme activity was defined as the consumed of 1.0 μM of acetyl-CoA per min at pH 8.0 at 30 °C. For determination of the feedback inhibition on acetyl-CoA carboxylase, C16-CoA was added into the cell-free extract of *Schizochytrium* sp. S31 at a final concentration of 50 or 100 μM. The activities of NADP^+^-dependent malic enzyme and acetyl-CoA carboxylase were normalized by the protein concentration of the supernatants.

### LC–MS metabolomic analysis

The LC–MS metabolomic analysis was carried out based on the method described previously [60]. Briefly, approximately 5.0 OD (OD_660_) of cells were harvested at 48 and 72 h by centrifugation at 8000×*g* for 5 min at room temperature (Eppendorf 5430R, Hamburg, Germany). The cells were immediately frozen at − 80 °C before use. The cells were added into a 900 μL of solution containing MeOH/H_2_O (v/v, 8:2, − 80 °C), quickly frozen in liquid nitrogen, and then thawed in room temperature. The samples were then frozen-thawed for three times. The supernatant was harvested by centrifugation at 15,000×*g* for 5 min at − 4 °C. The sediments were re-suspended in 500 μL of 80:20 MeOH/H_2_O (− 80 °C) and then repeated the above extraction process. The supernatant of the second extraction was mixed with the first, and the solvents were removed using a vacuum concentrator system (ZLS-1, Hunan, China). The sediment was resolved in 100 μL of distilled water. LC–MS analysis was performed on an Agilent 1260 series binary HPLC system (Agilent Technologies, Waldbronn, Germany) using a SYnergi Hydro-RP (C18) 150 mm × 2.0 mm I.D., 4 μm 80 Å particles column (Phenomenex, Torrance, CA, USA), coupled to an Agilent 6410 triple quadrupole mass analyser equipped with an electrospray ionization source. An aqueous solution containing 10 mM tributylamine and a 100% methanol (HPLC grade) solution were used as mobile phase A and B, respectively. The flow rate was set at 0.2 mL/min. The MS was operated in negative mode for multiple reaction monitoring development, method optimization, and sample analysis. Sample injected volume was 10 μL; capillary voltage was 4000 V; nebulizer gas flow rate was 10 L/min; nebulizer gas pressure was 50 psi; nitrogen nebulizer gas temperature was 300 °C. Data were obtained using Agilent Mass Hunter workstation LC/QQQ acquisition software (version B.04.01) and chromatographic peaks were subsequently integrated via Agilent Qualitative Analysis software (version B.04.00). Metabolomic data were normalized by interior control and cell number, and then subjected to the principal component analysis performed by SIMCA-P 11.5 software. Heatmaps of metabolomic data were created using the MultiExperiment Viewer software. For measuring the intracellular propionyl-CoA concentration, approximately 5.0 OD (OD_660_) of cells were harvested at 48 and 72 h, and the exaction and analysis methods followed the LC–MS metabolomic analysis described above. The standard propionyl-CoA purchased from Sigma (St. Louis, MO, USA) was used for determination of the standard curve of propionyl-CoA. The intracellular propionyl-CoA concentration was normalized by the value of OD_660_, which can be used for representing the biomass concentration in *Schizochytrium* [29].

### Statistical analysis

All experiments were conducted with at least three biological replicates. Statistical analyses were performed using two-tailed Student’s *t* tests, and *p* < 0.05 was considered indicative of statistical significance.

## Additional files

**Additional file 1: Fig. S1.** Comparison of acetyl-CoA carboxylase activity after adding C16-CoA into cell-free extract of *Schizochytrium* sp. S31. 0 represents adding 0 μM of C16-CoA; 50 represents adding 50 μM of C16-CoA; 100 represents adding 100 μM of C16-CoA. * indicated *p* < 0.05. ** indicated *p* < 0.01. **Fig. S2.** Expression of a codon-optimized *ELO3* gene from *Mortierella alpina* in *Schizochytrium*. **A**) Schematic map of the *ELO3* gene expression cassette, with the indicated primers. 1: 18s upstream; 2: CaMV 35s promoter; 3: *ELO3* gene; 4: Nos terminator; 5: α tubulin promoter from *Schizochytrium*; 6: bleomycin resistance gene; 7: CYC1 terminator; 8: 18s downstream; **B**) Genomic PCR detection with primer 1 and primer 2. M: 1 kb marker from Vazyme company; 1: S-E strain; 2: wild type. **C**) RT-PCR detection with primer 3 and primer 4. M: 1 kb marker from Thermo Scientific company; 1: S-E strain; 2: wild type. **Fig. S3.** Expression of a malic enzyme gene from *C*. *cohnii* in *Schizochytrium*. **A**) Schematic map of the malic enzyme expression cassette, with the indicated primers. 1: 18s upstream; 2: CaMV 35s promoter; 3: neomycin resistance gene; 4: CaMV poly(A) terminator; 5: malic enzyme gene; 6: 18s downstream; **B**) Genomic PCR detection with primer 5 and primer 6. M: 1kb marker; 1: water; 2: wild type; 3: S-M strain; **C**) RT-PCR detection with primer 7 and primer 8. 1: water; 2: wild type; 3 S-M strain. **Fig. S4.** Malic enzyme activity and NADPH content in the wild type and S-M strains. **A**) The malic enzyme activity. Blue: wild type; Purple: S-M strain. * indicated *p* < 0.05; **B**) NADPH content. Blue: wild type; Purple: S-M strain. * indicated *p* < 0.05, **indicated *p* < 0.01. **Fig. S5.** Expression of a codon-optimized *ELO3* gene from *M*. *alpina* in S-M strain. **A**) Schematic map of the *ELO3* gene expression cassette, with the indicated primers. 1: CaMV 35s promoter; 2: *ELO3* gene; 3: Nos terminator; 4: α tubulin promoter from *Schizochytrium*; 5: bleomycin resistance gene 6: CYC1 terminator; **B**) Genomic PCR detection with primer 1 and primer 2. M: 1 kb marker; 1: S-M-E strain; 2: S-M strain; **C**) RT-PCR detection with primer 3 and primer 4. M: 1 kb marker; 1: S-M strain; 2: S-M-E strain. **Fig. S6.** MS spectrums of methyl fatty acids detected among strains. **Fig. S7.** Principal component analysis of LC–MS targeted metabolomics of S-M and S-M-E strains. **A**) 48 h; **B**) 72 h. Purple: S-M strain; Green: S-M-E strain.

**Additional file 2: Table S1.** Fatty acids profiles in the wild type, S-E, S-M and S-M-E strains. **Table S2.** LC–MS metabolomics data sets of S-M and S-M-E strains. **Table S3.** The production cost of DHA and OCFAs in the wild type and S-M-E strains. **Table S4.** Primers used in this study.

**Additional file 3: File S1.** The sequence of codon-optimized *ELO3* gene from *Mortierella alpina*.

## Data Availability

All data generated or analyzed during this study are included in this published article and its additional files.

## Abbreviations

ACC: acetyl-CoA carboxylase
Ac-CoA: acetyl-CoA
ADP: adenosine diphosphate
AKG: 2-oxoglutaric acid
ALA: alanine
AMP: adenosine monophosphate
ATP: adenosine triphosphate
ASP: aspartic acid
C14: C14:0
C16: C16:0
C18: C18:0 and C18:1
CIT: citrate
Citric: citric acid
DHA: docosahexaenoic acid
FAS: fatty acid synthase
F6P: fructose 6-phosphate
FBP: fructose 1,6-bisphosphate
FUM: fumaric acid
G6P: glucose-6-phosphate
GAP: glyceraldehyde 3-phosphate
GLU: glutamic acid
LEU: isoleucine
LYS: lysine
MAL: malate
ME: malic enzyme
NADPH: nicotinamide adenine dinucleotide phosphate
NADP+: oxidized form of nicotinamide adenine dinucleotide phosphate
NAD+: nicotinamide adenine dinucleotide
OCFAs: odd-chain fatty acids
OXA: oxaloacetic acid
3-PG: 3-phosphoglyceric acid
6PG: 6-phosphogluconate
PEP: phosphoenolpyruvic acid
PHE: phenylalanine
PKS: polyketide synthase
PYR: pyruvate
Ri5P: ribose 5-phosphate
SER: serine
SUM: succinic acid
X5P: xylulose 5-phosphate
VAL: valine
