# Supplementary figures and images for: Metabolic engineering to enhance biosynthesis of both docosahexaenoic acid and odd-chain fatty acids in Schizochytrium sp. S31
Source: Biotechnol Biofuels. 2019 Jun 8;12:141. doi: 10.1186/s13068-019-1484-x (PMC6555965; doi:10.1186/s13068-019-1484-x)

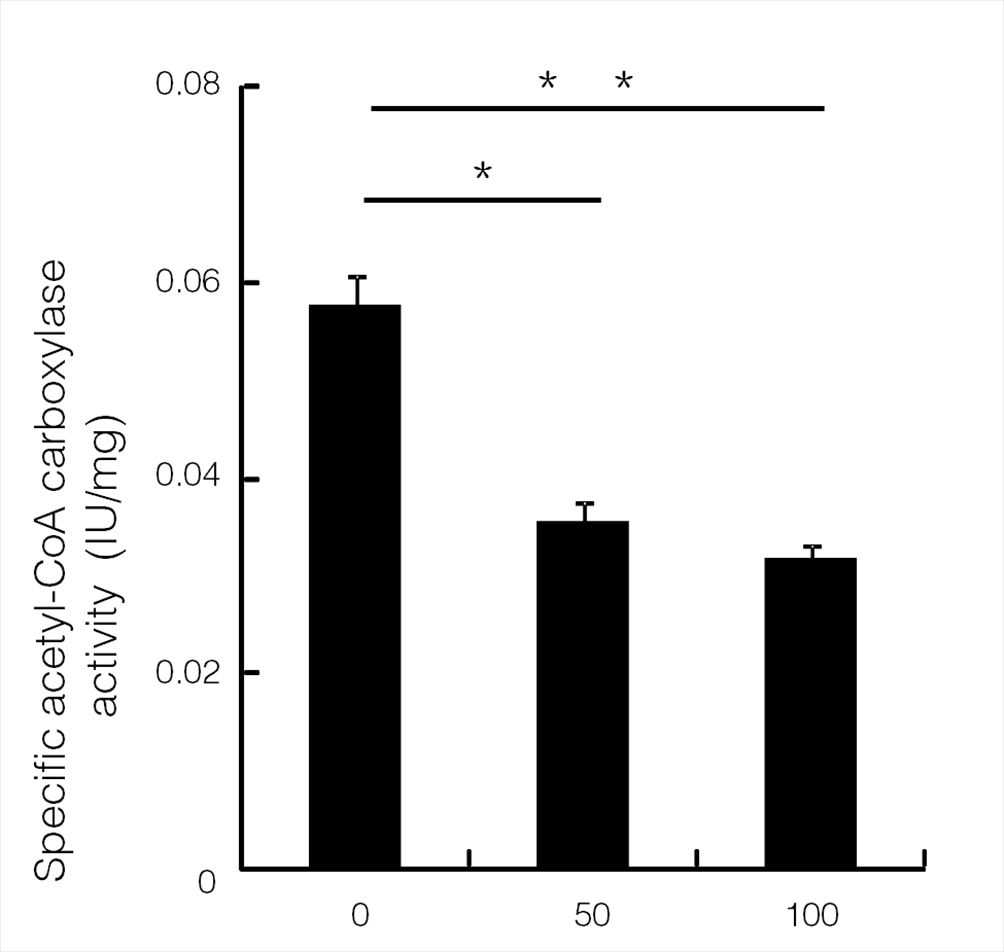

Supplement: Supplementary file 1 — Additional file 1: Fig. S1. Comparison of acetyl-CoA carboxylase activity after adding C16-CoA into cell-free extract of Schizochytrium sp. S31. 0 represents adding 0 μM of C16-CoA; 50 represents adding 50 μM of C16-CoA; 100 represents adding 100 μM of C16-CoA. * indicated p < 0.05. ** indicated p < 0.01. Fig. S2. Expression of a codon-optimized ELO3 gene from Mortierella alpina in Schizochytrium. A) Schematic map of the ELO3 gene expression cassette, with the indicated primers. 1: 18s upstream; 2: CaMV 35s promoter; 3: ELO3 gene; 4: Nos terminator; 5: α tubulin promoter from Schizochytrium; 6: bleomycin resistance gene; 7: CYC1 terminator; 8: 18s downstream; B) Genomic PCR detection with primer 1 and primer 2. M: 1 kb marker from Vazyme company; 1: S-E strain; 2: wild type. C) RT-PCR detection with primer 3 and primer 4. M: 1 kb marker from Thermo Scientific company; 1: S-E strain; 2: wild type. Fig. S3. Expression of a malic enzyme gene from C. cohnii in Schizochytrium. A) Schematic map of the malic enzyme expression cassette, with the indicated primers. 1: 18s upstream; 2: CaMV 35s promoter; 3: neomycin resistance gene; 4: CaMV poly(A) terminator; 5: malic enzyme gene; 6: 18s downstream; B) Genomic PCR detection with primer 5 and primer 6. M: 1kb marker; 1: water; 2: wild type; 3: S-M strain; C) RT-PCR detection with primer 7 and primer 8. 1: water; 2: wild type; 3 S-M strain. Fig. S4. Malic enzyme activity and NADPH content in the wild type and S-M strains. A) The malic enzyme activity. Blue: wild type; Purple: S-M strain. * indicated p < 0.05; B) NADPH content. Blue: wild type; Purple: S-M strain. * indicated p < 0.05, **indicated p < 0.01. Fig. S5. Expression of a codon-optimized ELO3 gene from M. alpina in S-M strain. A) Schematic map of the ELO3 gene expression cassette, with the indicated primers. 1: CaMV 35s promoter; 2: ELO3 gene; 3: Nos terminator; 4: α tubulin promoter from Schizochytrium; 5: bleomycin resistance gene 6: CYC1 terminator; B) Genomic PCR [file 13068_2019_1484_MOESM1_ESM.zip › Figure S1.tif]

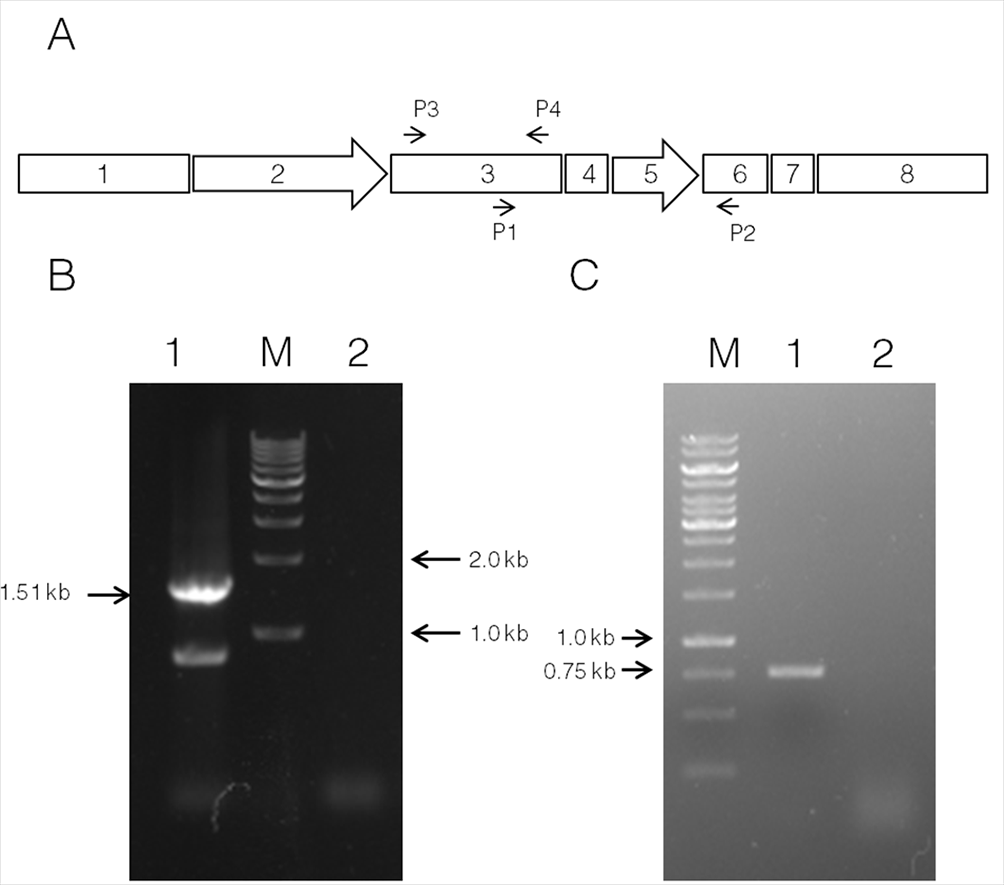

Supplement: Supplementary file 1 — Additional file 1: Fig. S1. Comparison of acetyl-CoA carboxylase activity after adding C16-CoA into cell-free extract of Schizochytrium sp. S31. 0 represents adding 0 μM of C16-CoA; 50 represents adding 50 μM of C16-CoA; 100 represents adding 100 μM of C16-CoA. * indicated p < 0.05. ** indicated p < 0.01. Fig. S2. Expression of a codon-optimized ELO3 gene from Mortierella alpina in Schizochytrium. A) Schematic map of the ELO3 gene expression cassette, with the indicated primers. 1: 18s upstream; 2: CaMV 35s promoter; 3: ELO3 gene; 4: Nos terminator; 5: α tubulin promoter from Schizochytrium; 6: bleomycin resistance gene; 7: CYC1 terminator; 8: 18s downstream; B) Genomic PCR detection with primer 1 and primer 2. M: 1 kb marker from Vazyme company; 1: S-E strain; 2: wild type. C) RT-PCR detection with primer 3 and primer 4. M: 1 kb marker from Thermo Scientific company; 1: S-E strain; 2: wild type. Fig. S3. Expression of a malic enzyme gene from C. cohnii in Schizochytrium. A) Schematic map of the malic enzyme expression cassette, with the indicated primers. 1: 18s upstream; 2: CaMV 35s promoter; 3: neomycin resistance gene; 4: CaMV poly(A) terminator; 5: malic enzyme gene; 6: 18s downstream; B) Genomic PCR detection with primer 5 and primer 6. M: 1kb marker; 1: water; 2: wild type; 3: S-M strain; C) RT-PCR detection with primer 7 and primer 8. 1: water; 2: wild type; 3 S-M strain. Fig. S4. Malic enzyme activity and NADPH content in the wild type and S-M strains. A) The malic enzyme activity. Blue: wild type; Purple: S-M strain. * indicated p < 0.05; B) NADPH content. Blue: wild type; Purple: S-M strain. * indicated p < 0.05, **indicated p < 0.01. Fig. S5. Expression of a codon-optimized ELO3 gene from M. alpina in S-M strain. A) Schematic map of the ELO3 gene expression cassette, with the indicated primers. 1: CaMV 35s promoter; 2: ELO3 gene; 3: Nos terminator; 4: α tubulin promoter from Schizochytrium; 5: bleomycin resistance gene 6: CYC1 terminator; B) Genomic PCR [file 13068_2019_1484_MOESM1_ESM.zip › Figure S2.tif]

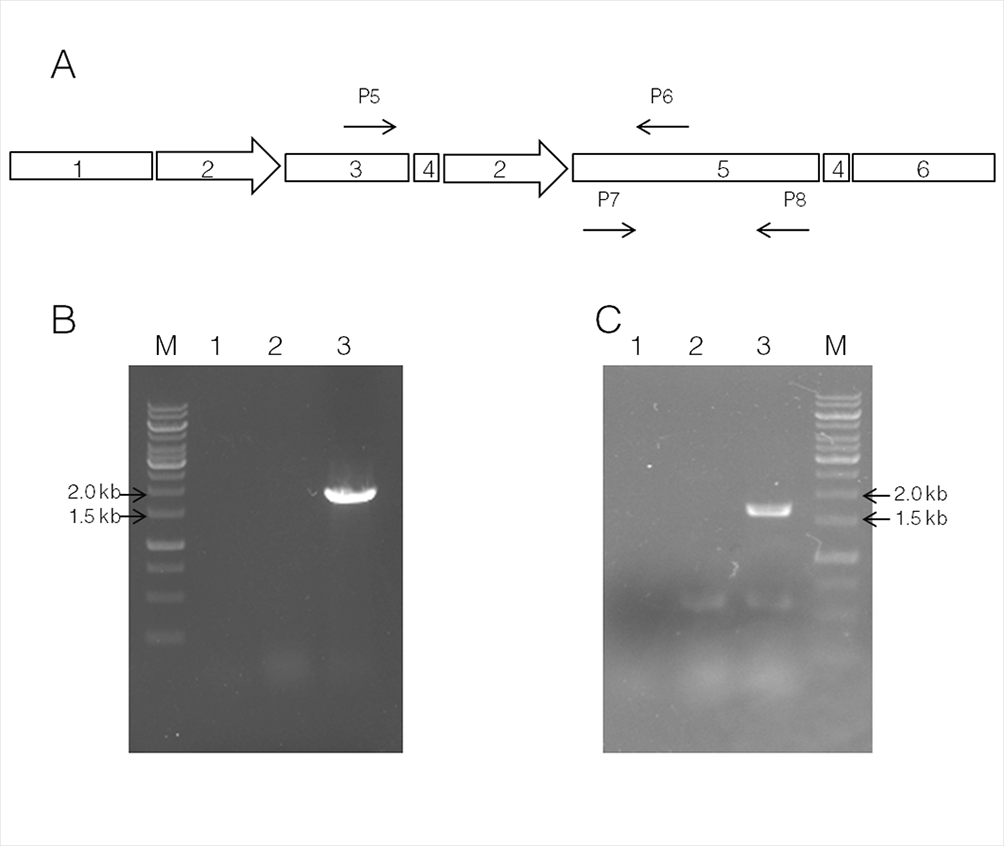

Supplement: Supplementary file 1 — Additional file 1: Fig. S1. Comparison of acetyl-CoA carboxylase activity after adding C16-CoA into cell-free extract of Schizochytrium sp. S31. 0 represents adding 0 μM of C16-CoA; 50 represents adding 50 μM of C16-CoA; 100 represents adding 100 μM of C16-CoA. * indicated p < 0.05. ** indicated p < 0.01. Fig. S2. Expression of a codon-optimized ELO3 gene from Mortierella alpina in Schizochytrium. A) Schematic map of the ELO3 gene expression cassette, with the indicated primers. 1: 18s upstream; 2: CaMV 35s promoter; 3: ELO3 gene; 4: Nos terminator; 5: α tubulin promoter from Schizochytrium; 6: bleomycin resistance gene; 7: CYC1 terminator; 8: 18s downstream; B) Genomic PCR detection with primer 1 and primer 2. M: 1 kb marker from Vazyme company; 1: S-E strain; 2: wild type. C) RT-PCR detection with primer 3 and primer 4. M: 1 kb marker from Thermo Scientific company; 1: S-E strain; 2: wild type. Fig. S3. Expression of a malic enzyme gene from C. cohnii in Schizochytrium. A) Schematic map of the malic enzyme expression cassette, with the indicated primers. 1: 18s upstream; 2: CaMV 35s promoter; 3: neomycin resistance gene; 4: CaMV poly(A) terminator; 5: malic enzyme gene; 6: 18s downstream; B) Genomic PCR detection with primer 5 and primer 6. M: 1kb marker; 1: water; 2: wild type; 3: S-M strain; C) RT-PCR detection with primer 7 and primer 8. 1: water; 2: wild type; 3 S-M strain. Fig. S4. Malic enzyme activity and NADPH content in the wild type and S-M strains. A) The malic enzyme activity. Blue: wild type; Purple: S-M strain. * indicated p < 0.05; B) NADPH content. Blue: wild type; Purple: S-M strain. * indicated p < 0.05, **indicated p < 0.01. Fig. S5. Expression of a codon-optimized ELO3 gene from M. alpina in S-M strain. A) Schematic map of the ELO3 gene expression cassette, with the indicated primers. 1: CaMV 35s promoter; 2: ELO3 gene; 3: Nos terminator; 4: α tubulin promoter from Schizochytrium; 5: bleomycin resistance gene 6: CYC1 terminator; B) Genomic PCR [file 13068_2019_1484_MOESM1_ESM.zip › Figure S3.tif]

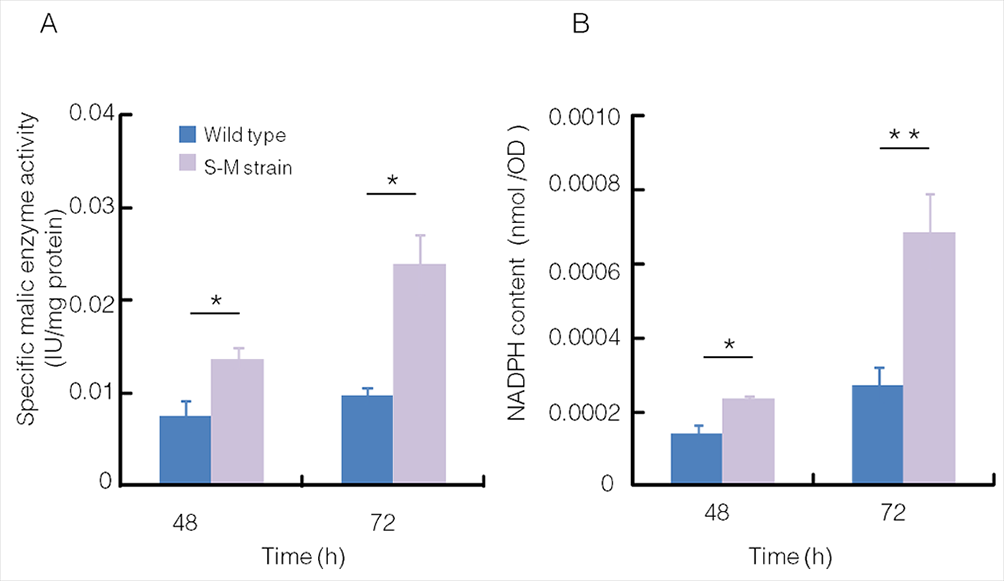

Supplement: Supplementary file 1 — Additional file 1: Fig. S1. Comparison of acetyl-CoA carboxylase activity after adding C16-CoA into cell-free extract of Schizochytrium sp. S31. 0 represents adding 0 μM of C16-CoA; 50 represents adding 50 μM of C16-CoA; 100 represents adding 100 μM of C16-CoA. * indicated p < 0.05. ** indicated p < 0.01. Fig. S2. Expression of a codon-optimized ELO3 gene from Mortierella alpina in Schizochytrium. A) Schematic map of the ELO3 gene expression cassette, with the indicated primers. 1: 18s upstream; 2: CaMV 35s promoter; 3: ELO3 gene; 4: Nos terminator; 5: α tubulin promoter from Schizochytrium; 6: bleomycin resistance gene; 7: CYC1 terminator; 8: 18s downstream; B) Genomic PCR detection with primer 1 and primer 2. M: 1 kb marker from Vazyme company; 1: S-E strain; 2: wild type. C) RT-PCR detection with primer 3 and primer 4. M: 1 kb marker from Thermo Scientific company; 1: S-E strain; 2: wild type. Fig. S3. Expression of a malic enzyme gene from C. cohnii in Schizochytrium. A) Schematic map of the malic enzyme expression cassette, with the indicated primers. 1: 18s upstream; 2: CaMV 35s promoter; 3: neomycin resistance gene; 4: CaMV poly(A) terminator; 5: malic enzyme gene; 6: 18s downstream; B) Genomic PCR detection with primer 5 and primer 6. M: 1kb marker; 1: water; 2: wild type; 3: S-M strain; C) RT-PCR detection with primer 7 and primer 8. 1: water; 2: wild type; 3 S-M strain. Fig. S4. Malic enzyme activity and NADPH content in the wild type and S-M strains. A) The malic enzyme activity. Blue: wild type; Purple: S-M strain. * indicated p < 0.05; B) NADPH content. Blue: wild type; Purple: S-M strain. * indicated p < 0.05, **indicated p < 0.01. Fig. S5. Expression of a codon-optimized ELO3 gene from M. alpina in S-M strain. A) Schematic map of the ELO3 gene expression cassette, with the indicated primers. 1: CaMV 35s promoter; 2: ELO3 gene; 3: Nos terminator; 4: α tubulin promoter from Schizochytrium; 5: bleomycin resistance gene 6: CYC1 terminator; B) Genomic PCR [file 13068_2019_1484_MOESM1_ESM.zip › Figure S4.tif]

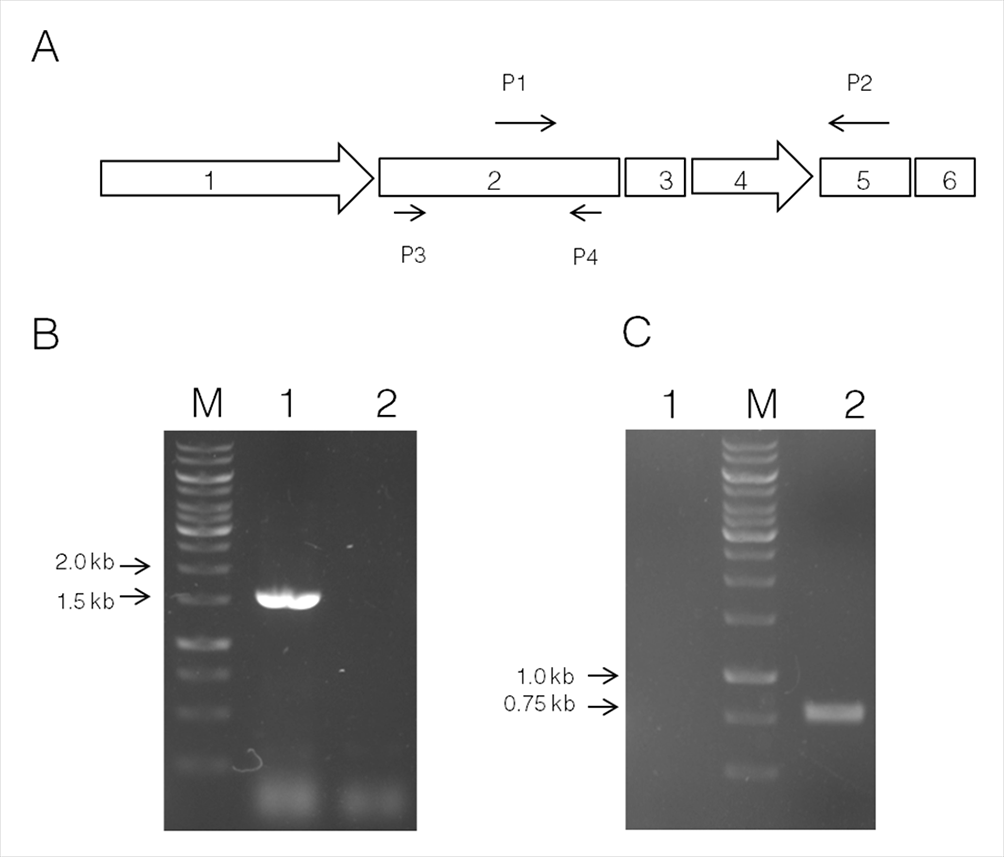

Supplement: Supplementary file 1 — Additional file 1: Fig. S1. Comparison of acetyl-CoA carboxylase activity after adding C16-CoA into cell-free extract of Schizochytrium sp. S31. 0 represents adding 0 μM of C16-CoA; 50 represents adding 50 μM of C16-CoA; 100 represents adding 100 μM of C16-CoA. * indicated p < 0.05. ** indicated p < 0.01. Fig. S2. Expression of a codon-optimized ELO3 gene from Mortierella alpina in Schizochytrium. A) Schematic map of the ELO3 gene expression cassette, with the indicated primers. 1: 18s upstream; 2: CaMV 35s promoter; 3: ELO3 gene; 4: Nos terminator; 5: α tubulin promoter from Schizochytrium; 6: bleomycin resistance gene; 7: CYC1 terminator; 8: 18s downstream; B) Genomic PCR detection with primer 1 and primer 2. M: 1 kb marker from Vazyme company; 1: S-E strain; 2: wild type. C) RT-PCR detection with primer 3 and primer 4. M: 1 kb marker from Thermo Scientific company; 1: S-E strain; 2: wild type. Fig. S3. Expression of a malic enzyme gene from C. cohnii in Schizochytrium. A) Schematic map of the malic enzyme expression cassette, with the indicated primers. 1: 18s upstream; 2: CaMV 35s promoter; 3: neomycin resistance gene; 4: CaMV poly(A) terminator; 5: malic enzyme gene; 6: 18s downstream; B) Genomic PCR detection with primer 5 and primer 6. M: 1kb marker; 1: water; 2: wild type; 3: S-M strain; C) RT-PCR detection with primer 7 and primer 8. 1: water; 2: wild type; 3 S-M strain. Fig. S4. Malic enzyme activity and NADPH content in the wild type and S-M strains. A) The malic enzyme activity. Blue: wild type; Purple: S-M strain. * indicated p < 0.05; B) NADPH content. Blue: wild type; Purple: S-M strain. * indicated p < 0.05, **indicated p < 0.01. Fig. S5. Expression of a codon-optimized ELO3 gene from M. alpina in S-M strain. A) Schematic map of the ELO3 gene expression cassette, with the indicated primers. 1: CaMV 35s promoter; 2: ELO3 gene; 3: Nos terminator; 4: α tubulin promoter from Schizochytrium; 5: bleomycin resistance gene 6: CYC1 terminator; B) Genomic PCR [file 13068_2019_1484_MOESM1_ESM.zip › Figure S5.tif]

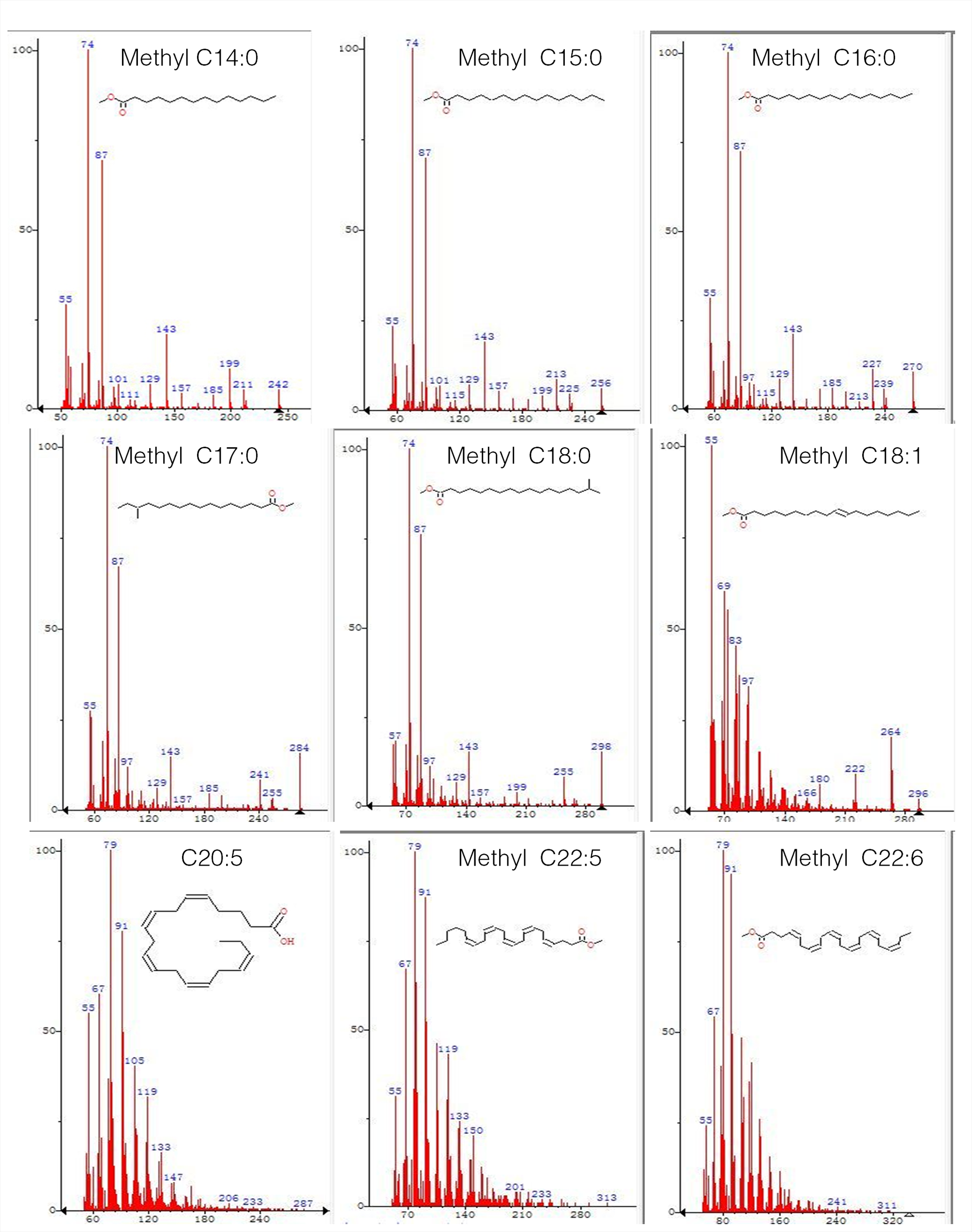

Supplement: Supplementary file 1 — Additional file 1: Fig. S1. Comparison of acetyl-CoA carboxylase activity after adding C16-CoA into cell-free extract of Schizochytrium sp. S31. 0 represents adding 0 μM of C16-CoA; 50 represents adding 50 μM of C16-CoA; 100 represents adding 100 μM of C16-CoA. * indicated p < 0.05. ** indicated p < 0.01. Fig. S2. Expression of a codon-optimized ELO3 gene from Mortierella alpina in Schizochytrium. A) Schematic map of the ELO3 gene expression cassette, with the indicated primers. 1: 18s upstream; 2: CaMV 35s promoter; 3: ELO3 gene; 4: Nos terminator; 5: α tubulin promoter from Schizochytrium; 6: bleomycin resistance gene; 7: CYC1 terminator; 8: 18s downstream; B) Genomic PCR detection with primer 1 and primer 2. M: 1 kb marker from Vazyme company; 1: S-E strain; 2: wild type. C) RT-PCR detection with primer 3 and primer 4. M: 1 kb marker from Thermo Scientific company; 1: S-E strain; 2: wild type. Fig. S3. Expression of a malic enzyme gene from C. cohnii in Schizochytrium. A) Schematic map of the malic enzyme expression cassette, with the indicated primers. 1: 18s upstream; 2: CaMV 35s promoter; 3: neomycin resistance gene; 4: CaMV poly(A) terminator; 5: malic enzyme gene; 6: 18s downstream; B) Genomic PCR detection with primer 5 and primer 6. M: 1kb marker; 1: water; 2: wild type; 3: S-M strain; C) RT-PCR detection with primer 7 and primer 8. 1: water; 2: wild type; 3 S-M strain. Fig. S4. Malic enzyme activity and NADPH content in the wild type and S-M strains. A) The malic enzyme activity. Blue: wild type; Purple: S-M strain. * indicated p < 0.05; B) NADPH content. Blue: wild type; Purple: S-M strain. * indicated p < 0.05, **indicated p < 0.01. Fig. S5. Expression of a codon-optimized ELO3 gene from M. alpina in S-M strain. A) Schematic map of the ELO3 gene expression cassette, with the indicated primers. 1: CaMV 35s promoter; 2: ELO3 gene; 3: Nos terminator; 4: α tubulin promoter from Schizochytrium; 5: bleomycin resistance gene 6: CYC1 terminator; B) Genomic PCR [file 13068_2019_1484_MOESM1_ESM.zip › Figure S6.tif]

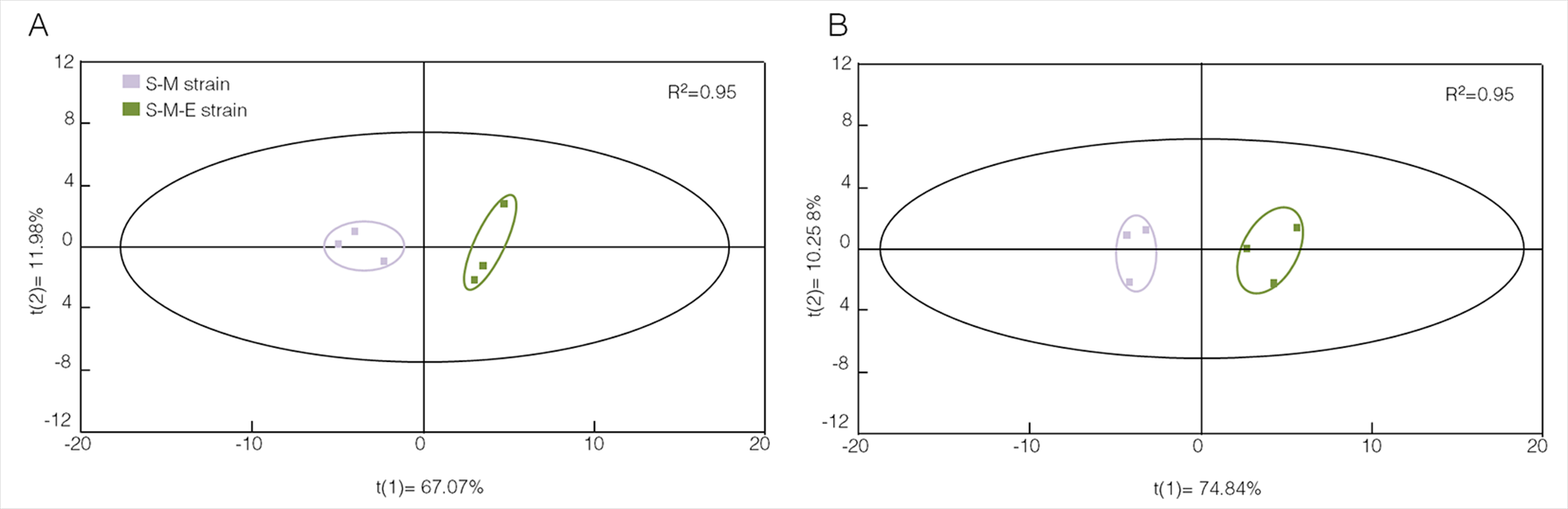

Supplement: Supplementary file 1 — Additional file 1: Fig. S1. Comparison of acetyl-CoA carboxylase activity after adding C16-CoA into cell-free extract of Schizochytrium sp. S31. 0 represents adding 0 μM of C16-CoA; 50 represents adding 50 μM of C16-CoA; 100 represents adding 100 μM of C16-CoA. * indicated p < 0.05. ** indicated p < 0.01. Fig. S2. Expression of a codon-optimized ELO3 gene from Mortierella alpina in Schizochytrium. A) Schematic map of the ELO3 gene expression cassette, with the indicated primers. 1: 18s upstream; 2: CaMV 35s promoter; 3: ELO3 gene; 4: Nos terminator; 5: α tubulin promoter from Schizochytrium; 6: bleomycin resistance gene; 7: CYC1 terminator; 8: 18s downstream; B) Genomic PCR detection with primer 1 and primer 2. M: 1 kb marker from Vazyme company; 1: S-E strain; 2: wild type. C) RT-PCR detection with primer 3 and primer 4. M: 1 kb marker from Thermo Scientific company; 1: S-E strain; 2: wild type. Fig. S3. Expression of a malic enzyme gene from C. cohnii in Schizochytrium. A) Schematic map of the malic enzyme expression cassette, with the indicated primers. 1: 18s upstream; 2: CaMV 35s promoter; 3: neomycin resistance gene; 4: CaMV poly(A) terminator; 5: malic enzyme gene; 6: 18s downstream; B) Genomic PCR detection with primer 5 and primer 6. M: 1kb marker; 1: water; 2: wild type; 3: S-M strain; C) RT-PCR detection with primer 7 and primer 8. 1: water; 2: wild type; 3 S-M strain. Fig. S4. Malic enzyme activity and NADPH content in the wild type and S-M strains. A) The malic enzyme activity. Blue: wild type; Purple: S-M strain. * indicated p < 0.05; B) NADPH content. Blue: wild type; Purple: S-M strain. * indicated p < 0.05, **indicated p < 0.01. Fig. S5. Expression of a codon-optimized ELO3 gene from M. alpina in S-M strain. A) Schematic map of the ELO3 gene expression cassette, with the indicated primers. 1: CaMV 35s promoter; 2: ELO3 gene; 3: Nos terminator; 4: α tubulin promoter from Schizochytrium; 5: bleomycin resistance gene 6: CYC1 terminator; B) Genomic PCR [file 13068_2019_1484_MOESM1_ESM.zip › Figure S7.tif]
