# Supplementary material for: Metabolic engineering to enhance biosynthesis of both docosahexaenoic acid and odd-chain fatty acids in Schizochytrium sp. S31
Source: Biotechnol Biofuels. 2019 Jun 8;12:141. doi: 10.1186/s13068-019-1484-x (PMC6555965; doi:10.1186/s13068-019-1484-x)
Supplement: Supplementary file 3 — Additional file 3: File S1. The sequence of codon-optimized ELO3 gene from Mortierella alpina. [file 13068_2019_1484_MOESM3_ESM.docx]

> The sequence of codon-optimized *ELO3* gene from *Mortierella alpina*

ATGGAGTCCGGCCCTATGCCTGCCGGCATCCCCTTCCCTGAGTACTACGACTTTTTCATGGACTGGAAGACCCCCCTCGCCATCGCTGCCACCTACACCGTCGCTGTCGGCCTCTTCAACCCCAAGGTCGGCAAGGTCTCGCGCGTGGTCGCCAAGTCGGCTAACGCCAAGCCGGCTGAGCGCACCCAGTCCGGCGCCGCCATGACCGCCTTTGTCTTTGTCCACAACCTCATCCTCTGCGTGTACTCCGGCATCACCTTCTACCACATGTTCCCGGCCATGGTCAAGAACTTTCGCACCCACACCCTCCACGAGGCCTACTGCGATACGGACCAGAGCCTCTGGAACAACGCCCTCGGCTACTGGGGCTACCTCTTCTACCTTTCCAAGTTTTACGAGGTCATTGACACCATCATCATCATCCTCAAGGGCCGCCGCTCGTCCCTCCTCCAGACCTACCACCACGCCGGCGCTATGATCACCATGTGGTCCGGCATCAACTACCAGGCCACCCCCATTTGGATTTTTGTCGTCTTCAACTCGTTCATCCACACCATCATGTACTGTTACTACGCCTTCACCTCGATCGGCTTCCACCCCCCCGGCAAGAAGTACCTCACCTCCATGCAGATCACCCAGTTTCTCGTCGGCATCACCATCGCCGTCTCCTACCTCTTCGTCCCTGGCTGCATCCGCACCCCCGGTGCTCAGATGGCTGTCTGGATCAACGTCGGCTACCTCTTTCCCCTCACCTACCTCTTTGTGGATTTTGCCAAGCGCACCTACTCCAAGCGCACCGCCATCGCCGCTCAGAAGAAGGCCCAGTAA
